# The CXCL12/CXCR4 Signaling Pathway: A New Susceptibility Factor in Human Papillomavirus Pathogenesis

**DOI:** 10.1371/journal.ppat.1006039

**Published:** 2016-12-05

**Authors:** Floriane Meuris, Laetitia Carthagena, Agnieszka Jaracz-Ros, Françoise Gaudin, Pasquale Cutolo, Claire Deback, Yuezhen Xue, Françoise Thierry, John Doorbar, Françoise Bachelerie

**Affiliations:** 1 Inflammation Chemokines and Immunopathology, INSERM, Fac. de médecine—Univ Paris-Sud, Université Paris-Saclay, Clamart, France; 2 US31-UMS3679 -Plateforme PHIC, Institut Paris-Saclay d’Innovation Thérapeutique (IPSIT), Inserm, CNRS, Univ Paris-Sud, Université Paris-Saclay, Clamart, France; 3 Papillomavirus Regulation and Cancer, Institute of Medical Biology, A*STAR, Singapore; 4 Department of Pathology, University of Cambridge, Cambridge, United Kingdom; Penn State University School of Medicine, UNITED STATES

## Abstract

The productive human papillomavirus (HPV) life cycle is tightly linked to the differentiation and cycling of keratinocytes. Deregulation of these processes and stimulation of cell proliferation by the action of viral oncoproteins and host cell factors underlies HPV-mediated carcinogenesis. Severe HPV infections characterize the wart, hypogammaglobulinemia, infection, and myelokathexis (WHIM) immunodeficiency syndrome, which is caused by gain-of-function mutations in the CXCR4 receptor for the CXCL12 chemokine, one of which is CXCR4^1013^. We investigated whether CXCR4^1013^ interferes in the HPV18 life cycle in epithelial organotypic cultures. Expression of CXCR4^1013^ promoted stabilization of HPV oncoproteins, thus disturbing cell cycle progression and proliferation at the expense of the ordered expression of the viral genes required for virus production. Conversely, blocking CXCR4^1013^ function restored virus production and limited HPV-induced carcinogenesis. Thus, CXCR4 and its potential activation by genetic alterations in the course of the carcinogenic process can be considered as an important host factor for HPV carcinogenesis.

## Author Summary

Human papillomaviruses (HPV) are epitheliotropic tumor viruses causing mostly benign warts but that have developed strategies to establish persistent infections. Although host immune responses clear most infections, persistence of some HPV types causes ~5% of human cancers and severe pathogenesis in immunosuppressed individuals. How early events in HPV infection, determined by the interaction between viral and host proteins, might lead to viral persistence and pathogenesis is unknown. Here, we thought to investigate this issue by providing mechanistic insights into the selective susceptibility to HPV pathogenesis displayed by patients who are immunosuppressed as a consequence of mutations in the *CXCR4* gene encoding for the receptor of the CXCL12 chemokine (WHIM syndrome). We previously unraveled the existence of a general interplay between the CXCL12/CXCR4 axis and HPV, which is hijacked toward cell transformation upon expression of the CXCR4 mutant. Here, using three dimensional epithelial cell cultures to analyze the HPV life cycle, we found that the CXCR4 mutant promotes cell hyperproliferation and stabilization of viral oncoprotein expression at the expense of virus production. Our results, which identify CXCR4 as an important gatekeeper of keratinocyte proliferation and as a new susceptibility factor in HPV pathogenesis, may be translated into anti-viral and anti-cancer strategies.

## Introduction

Human papillomaviruses (HPVs) are a family of highly related non-enveloped epitheliotropic viruses that have co-evolved with their human host and developed powerful strategies to establish persistent infection [1]. Many reports have described HPVs as commensal viruses that can persist on healthy skin [2–4]. HPVs selectively infect basal keratinocytes of stratified epithelia and other discrete populations, including the cells located in the squamocolumnar junction of the cervix [5, 6]. These viruses undergo productive replication strictly in the terminally differentiated layers of the infected epithelium, and in most cases, cause no tissue damage or only benign warts [7]. Although host immune responses resolve most infections, instauration of persistent infections by the mucosal types of HPVs classified as high-risk, of which HPV16 and HPV18 are the most significant, is responsible for almost all cases of cervical carcinoma, a leading cause of cancer death in women. These viruses are also responsible for most anal cancers, as well as a fraction of vulval, vaginal, penile, and oropharyngeal cancers, causing nearly 5% of human cancers worldwide [8]. Cutaneous high-risk HPV types that normally persist without symptoms have also been associated with non-melanoma skin cancers in some rare genetic diseases or in immunosuppressed patients [9, 10]. The oncogenicity of high-risk HPVs appears in the context of asymptomatic persistent infections that can hinder host immune responses as a result of evasion and subversion strategies [11]. In support of this idea, clinical observations suggest that the frequency of HPV-associated cancers is increased in immunosuppressed patients [12, 13].

HPV-associated cancers are generally non-productive infections in which the viral E6 and E7 oncoproteins are abnormally expressed. In contrast, the timing and induction of E6 and E7 expression are tightly controlled during productive HPV infection, as the infected cells migrate towards the epithelial surface where the sequential appearance of the E4, L2, and L1 viral proteins allows virus release [14]. Although HPV oncoproteins are primarily responsible for the initiation and progression of cancer, only a small percentage of people infected with high-risk HPVs develop cancers. This indicates a contribution for host factors in HPV malignancy, although their mechanisms remain poorly understood [1].

One possible mechanism is that the changes in host cell signaling pathways that occur during disease progression arise from deregulated E6/E7 oncoprotein expression, which increases the risk of transformation [15]. HPV integration found in many HPV-positive carcinomas predominantly results in deregulated oncoprotein expression. Integration has also been associated with several recurrent genomic alterations and the elevated expression of host cell genes adjacent to the integration site [16]. Additionally, the oncoproteins of high-risk HPV types have been proposed to induce genomic instability through subversion of the cellular functions involved in DNA damage and repair responses [11, 17]. In addition, host factors might confer high susceptibility for the development of HPV-associated cancers, as cervical intraepithelial neoplasias and cancers related to carcinogenic HPV infection have been linked to infection of the cervical reserve cells [18] and/or the discrete population of cuboidal cells, which are located in the cervical squamocolumnar junction and express a unique panel of genes [5, 19]. Moreover, severe HPV-associated pathogenesis (e.g. persistent verrucosis, dysplasia, neoplasia, some cutaneous squamous cell cancers, and a high prevalence of genital cancer) is an underappreciated major manifestation of some primary immunodeficiencies and investigation of these clinical developments has provided clues to the host risk factors involved [20].

The wart, hypogammaglobulinemia, infection, and myelokathexis (WHIM) syndrome is associated with inherited gain-of-function mutations in the *CXCR4* gene that encodes a receptor for the CXCL12 chemokine [21]. Binding of CXCL12 to CXCR4 triggers typical activation of Gαi protein-dependent pathways of a chemokine receptor that are regulated in a timely manner by β-arrestins, which preclude further G protein activation (i.e., desensitization) and also link CXCR4 to additional signaling pathways involved in cytoskeleton reorganization and anti-apoptotic signaling [22, 23]. In WHIM, the CXCL12/CXCR4 signaling pathways manifest by abnormally increased and prolonged G protein- and β-arrestin-dependent responses associated with an impaired desensitization of CXCR4. Such dysfunction are responsible for the characteristic panleukopenia [24] in WHIM patients and likely also account for the severity of HPV disease through mechanisms that involve target cells and systemic immunity. From an immunological perspective, the unprecedented remission of HPV-induced warts in a WHIM patient after spontaneous partial inactivation of CXCR4 and subsequent restoration of immune function [25] suggests a role for myeloid cells in HPV life-cycle in support of the anomalies observed in WHIM patients’ myeloid cells [26]. However, whether it indicates that myeloid cells participate to host defense against HPV or, that they contribute to HPV-induced disease, when altered in WHIM patients, as reported in instances of chronic inflammation [27], remains unknown. Evidence for the involvement of the CXCL12/CXCR4 pair in the HPV life cycle arose from the abnormal and specific expression of CXCL12 observed in keratinocytes of HPV-productive skin or mucosal lesions regardless of whether patients suffer from WHIM [28]. Expression levels of CXCL12 and its receptors, which increase in keratinocytes as a consequence of HPV genome expression, generate an autocrine signaling loop essential for keratinocyte proliferation and migration. This interplay is involved in the WHIM-associated gain-of-function CXCR4 mutant, which confers transforming capacity on HPV18-immortalized keratinocytes in mice [29]. This further supports the hypothesis that dysfunction of the CXCL12/CXCR4 signaling pathway contribute to the pathogenesis of HPV-associated cancer.

However, the mechanism accounting for this process and whether it affects viral replication is not known. Here, we investigated a possible role for the WHIM-associated gain-of-function mutant receptor CXCR4^1013^ in the HPV life cycle using HPV18 in the context of three-dimensional organotypic raft cultures of keratinocytes, the sole replicative model for HPV [30]. Our data indicate that the CXCR4^1013^ mutant receptor shifts the HPV18 life cycle toward carcinogenesis, which is reflected in a viral gene expression pattern that favors oncoprotein stabilization. Blockade of either the CXCL12/CXCR4 axis or downstream effectors restored the productive HPV life cycle. Thus a main finding is that the WHIM-associated mutant CXCR4 receptor has a keratinocyte-intrinsic effect on HPV life cycle, supporting the idea that not all the effects of this mutation are mediated through dysregulation of the immune system. Besides, our results demonstrate an important function for the CXCL12/CXCR4 axis in the control of keratinocyte proliferation and its role in triggering transformation by HPV upon signaling imbalances resulting from cell hyperproliferation and elevated levels of oncoprotein-induced signaling.

## Results

### CXCR4^1013^ alters the productive HPV life cycle

**Fig 1:**
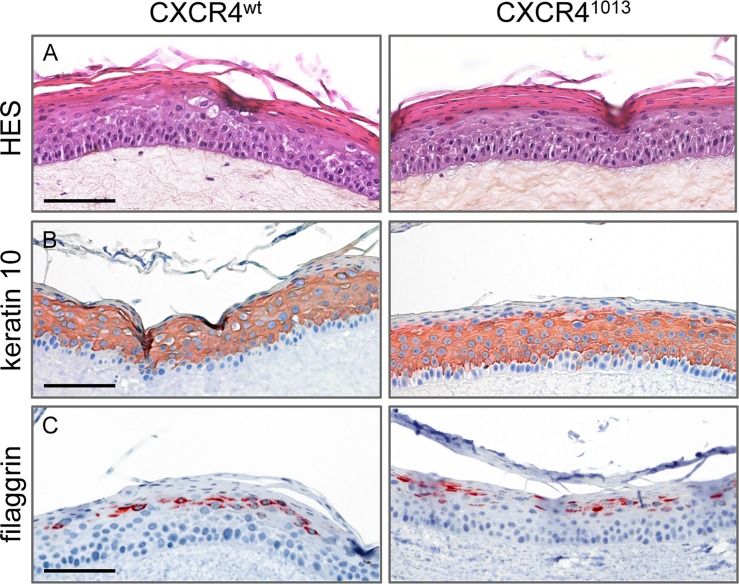
Expression of CXCR4^1013^ does not modify the architecture of raft cultures or the differentiation program of keratinocytes. (A) Representative HPV18-positive CXCR4^wt^ and CXCR4^1013^ raft culture sections stained with hematoxylin, eosin, and safran (HES) (A) or stained for keratin 10 (B) or filaggrin (C). Images are representative of three independent experiments. Scale bars = 100 μm.

**Fig 2:**
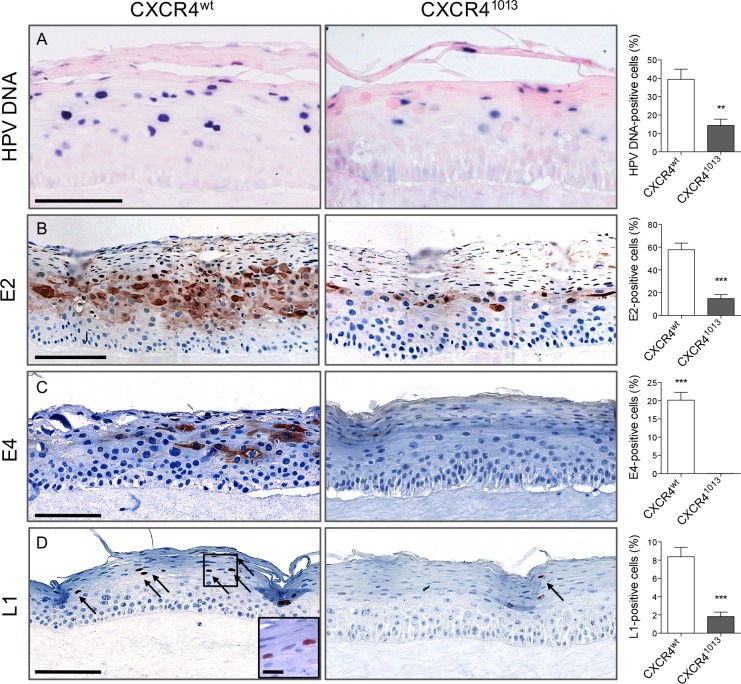
Abortive HPV18 infection in rafts cultures expressing CXCR4^1013^. Images of HPV18-positive CXCR4^wt^ and CXCR4^1013^ raft culture sections depicting HPV DNA by in situ hybridization (A) and HPV18-E2 (B), HPV18-E4 (C), and HPV18-L1 (D) by immunohistochemical staining. Quantifications of HPV DNA-, E2-, E4- and L1-positive cells from histological analyses are expressed as the percentage of positive cells out of the total number of epithelial cells. Values are means ± SEM. **p < 0.01, ***p < 0.001. Images are representative of three independent experiments. Scale bars = 100 μm, inset scale bar = 10 μm.

Expression of CXCR4^1013^ but not the wild-type CXCR4 receptor (CXCR4^wt^) confers primary human keratinocytes immortalized by HPV18 with transforming capacity, such that they develop solid tumors in nude mice [29]. We therefore explored a role for CXCR4^1013^ in the HPV vegetative cycle in experimental models. As HPV properly replicates only within stratified epithelium, we set up three-dimensional organotypic cultures (raft cultures) that form dermal and epidermal layers resembling those in skin. These cultures support the productive HPV life cycle because the infected epithelial cells differentiate during their migration towards the epithelial surface. We used spontaneously immortalized human keratinocytes (NIKS cells) that grow normally, and can undergo terminal differentiation when cultured in rafts [31]. In NIKS cells the expression levels of ectopically expressed wt and mutant CXCR4 receptors (CXCR4^wt^ or CXCR4^1013^) were in the same range at the RNA and protein levels (S1 Fig, panels A, B and C). Viral genome copy numbers were similar among the various NIKS cells in monolayer (i.e. expressing the endogenous CXCR4^wt^ solely or with either CXCR4^wt^ or CXCR4^1013^ ectopically expressed receptors) and were increased in the same range upon differentiation of NIKS cells in organotypic cultures (S2 Fig, panel A). Viral transcript levels (E6E7 and E2) were also similar in NIKS cells expressing the exogenous wt or mutant forms of the receptor (S2 Fig, panel B). The global architectures and subcellular topologies of raft cultures expressing CXCR4^1013^ or CXCR4^wt^ (CXCR4^1013^- or CXCR4^wt^-rafts) was apparently unchanged within CXCR4^1013^-expressing raft cultures (Fig 1, panel A) as well as the expression pattern of CXCR4 receptors (endogenous CXCR4 and ectopically expressed wt and mutant forms) in basal and supra-basal layers (S1 Fig, panel D). CXCR4^1013^- and CXCR4^wt^-rafts also exhibited normal expression patterns for keratin 10 in the spinous and granular layers, and the keratin filament-associated filaggrin protein in the granular layer, which are two prominent markers of epidermal differentiation (Fig 1, panels B-C). In contrast, replication of the viral genome was strongly diminished in CXCR4^1013^-raft cultures compared to that in CXCR4^wt^-rafts (Fig 2, panel A). Consistent with its role in HPV replication [32], production of the E2 viral protein was also lower in CXCR4^1013^-raft cultures (Fig 2, panel B). Production of viral proteins involved in the completion of the HPV life cycle in the upper epithelial layers, were also dramatically reduced (L1) or nearly undetectable (E4) in CXCR4^1013^-rafts compared to their levels in CXCR4^wt^-rafts (Fig 2, panels C-D). The E4 expression pattern in native NIKS raft cultures expressing endogenous CXCR4 (S3 Fig) was comparable to that observed in CXCR4^wt^-raft cultures (Fig 2, panel C), further supporting the relevance of the CXCR4^wt^-raft model. We have then searched for the biosynthesis of infectious viral particles, which is the final step in the HPV life cycle. We found that CXCR4^wt^-rafts are producing virions, which were infectious in an HaCaT cell infection assay as detected by the presence of the HPV spliced E1E4 transcript [33], while the CXCR4^1013^-rafts did not contain detectable infectious virions (S4 Fig). These data were correlated with the presence of koilocytic cells in the intermediate layers of the CXCR4^wt^-rafts (e.g. Fig 1, panels A-B). Collectively, the dramatic lowest production of E2, L1, and E4 proteins in CXCR4^1013^-rafts together with the absence of any detectable infectious virions strongly suggest that this cell environment restricts replication and the productive HPV life cycle.

### CXCR4^1013^ promotes oncogene expression

**Fig 3:**
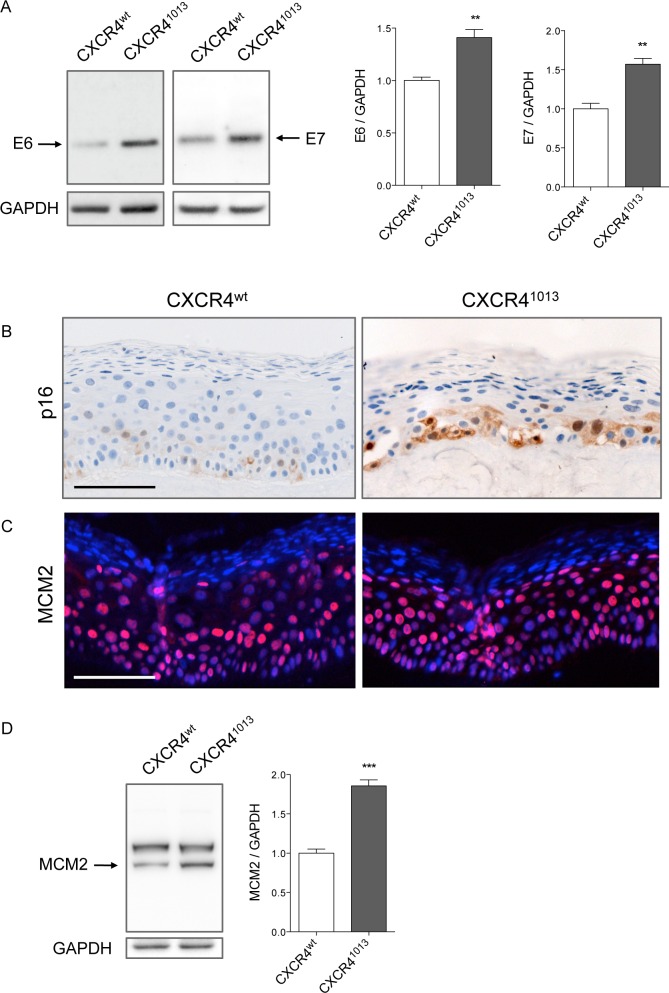
Expression of HPV oncoproteins and their surrogate markers is enhanced in CXCR4^1013^ rafts. (A) Western blots (left) and densitometric analyses (right) showing relative levels of HPV18-E6 and HPV18-E7 expression in HPV18-positive CXCR4^wt^ and CXCR4^1013^ rafts. E6 and E7 protein levels were normalized to GAPDH protein levels and arbitrarily set at 1 for CXCR4^wt^ rafts (staining controls are shown in S5 Fig). Values are means ± SEM. **p < 0.01. HPV18-positive CXCR4^wt^ and CXCR4^1013^ raft sections stained for p16 expression by immunohistochemistry (B) and for MCM2 expression by immunofluorescence (C). Detection of MCM2 expression by western blot (D). MCM2 protein levels were normalized to GAPDH protein levels and arbitrarily set at 1 for CXCR4^wt^ rafts. Means ± SEM are shown. ***p < 0.001. Images are representative of three independent experiments. Scale bars = 100 μm.

To gain further insight into the consequences of CXCR4^1013^ expression on HPV life cycle, we analyzed expression of the E6 and E7 viral oncogenes and their surrogate markers. Western blot analyses of raft cultures showed that E6 and E7 protein production in CXCR4^1013^-rafts was significantly higher than in CXCR4^wt^-rafts (Fig 3, panel A and control experiments for antibodies specificity in S5 Fig). Since we were unable to detect E7 and E6 proteins in raft cultures by immunohistochemistry because of high background, we used surrogate markers to investigate the expression of these proteins. E7 is known to disrupt proteins involved in cell cycle progression, resulting in substantial induction of a functionally inactive form of cyclin-dependent kinase inhibitor 2A (p16), which can be used as an indicator of deregulated E7-expression and HPV-associated dysplastic and neoplastic lesions [9]. The minichromosome maintenance (MCM) family of cell cycle proteins are essential for eukaryotic DNA replication, and MCM7 and MCM2 are widely used as molecular surrogates of E6/E7 expression and E6/E7-mediated cell cycle entry [34]. In agreement with this, MCM2 and p16 expression levels were higher in CXCR4^1013^-rafts than in CXCR4^wt^-rafts. Compared to the limited distribution of p16 staining in basal layers, MCM2 staining was more widespread and intense, and extended into the upper epithelial layers (Fig 3, panels B-C-D). The significantly higher levels of oncogene expression observed in CXCR4^1013^-rafts, were not paralleled by neither higher E6/E7 transcript levels nor changes in E2 transcripts levels (S6 Fig, panel A). This was consistent with our inability to detect any HPV genome integration (S6 Fig, panel B) or enhanced transcriptional activity of the HPV long control region (LCR) (S6 Fig, panel C) in CXCR4^1013^-rafts.

To determine whether the E6 and E7 oncoproteins were stabilized in CXCR4^1013^ cells, we quantified E6 and E7 protein levels over time in CXCR4^wt^- and CXCR4^1013^-expressing keratinocytes induced to differentiate in medium containing a high concentration of calcium. This model permits the activation of late events and the productive phase of the HPV life cycle after 48−96 h of culture [35]. In NIKS cells differentiated for 96 h in high-calcium medium, E6 and E7 protein decay after 2 h of cycloheximide treatment was lower in CXCR4^1013^ keratinocytes than in CXCR4^wt^ keratinocytes, resulting in higher relative levels of E6 and E7 in CXCR4^1013^ keratinocytes (S7 Fig, panel A). In contrast, in undifferentiated NIKS cells we observed no significant difference in the stability of oncogenes (S7 Fig, panel B). These results suggest that the viral oncoproteins were stabilized in the presence of CXCR4^1013^ in differentiated cells.

### HPV life cycle deregulation is associated with early markers of carcinogenesis

**Fig 4:**
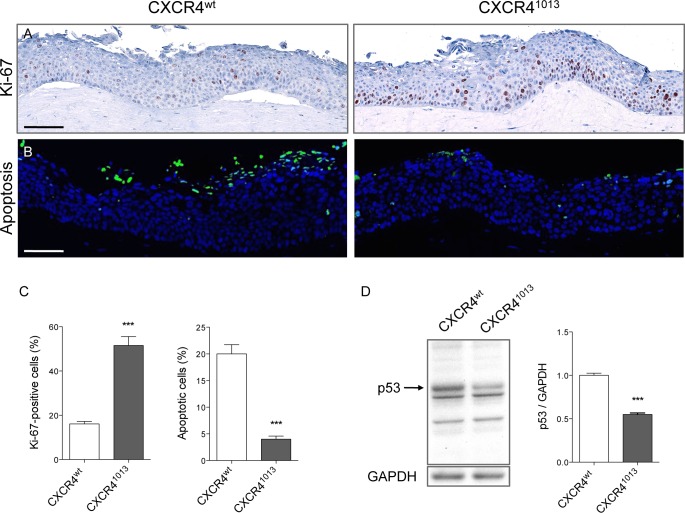
CXCR4^1013^ expression in raft cultures promotes keratinocyte proliferation and reduces apoptosis. (A) Detection of Ki-67 expression by immunohistochemical staining in proliferating HPV18-positive CXCR4^wt^ and CXCR4^1013^ raft culture sections. (B) Apoptotic cells (green) in HPV18-positive CXCR4^wt^ and CXCR4^1013^ rafts identified by TUNEL assay. Nuclei (blue) were counterstained with DAPI. Images are representative of three independent experiments. Scale bars = 100 μm. (C) Quantification of Ki-67-positive and apoptotic cells from the histological analyses. Results are expressed as the percentage of positive cells out of the total number of epithelial cells. Values are means ± SEM. ***p < 0.001. (D) Western blots (left) and densitometric analyses (right) showing relative levels of p53 in HPV18-positive CXCR4^wt^ and CXCR4^1013^ rafts. p53 protein levels were normalized to GAPDH protein levels and arbitrarily set at 1 for CXCR4^wt^ rafts. Values are means ± SEM. ***p < 0.001.

An elevated proliferation rate is an early step in viral oncogenesis [8]. The Ki-67 antigen, which is expressed in all phases of the cell cycle except in G0, is widely used to assess proliferation and represents a biomarker for cervical cancer [34]. Immunohistochemical analyses of Ki-67 in raft cultures indicated higher levels of cellular proliferation in the basal and suprabasal compartments of CXCR4^1013^ rafts than in those of CXCR4^wt^ rafts, and a higher overall proportion of (Ki-67-positive) cells in cycle (Fig 4 panels A, C). Keratinocyte proliferation is normally restricted to the basal layers in CXCR4^1013^-rafts in the absence of HPV infection (S8 Fig), indicating that CXCR4^1013^ does not deregulate cell proliferation on its own but rather acts synergistically with HPV. Given the capacity of E6 to promote degradation of the p53 protein, a gatekeeper of aberrant cell cycle progression involved in cell cycle arrest and apoptosis [36], we quantified apoptotic cell numbers in raft sections by TUNEL assay and p53 protein levels by western blot (Fig 4). There were fewer TUNEL-positive apoptotic cells in sections of CXCR4^1013^ rafts than in CXCR4^wt^ rafts suggesting that the overall proportion of apoptotic cells can significantly lower in CXCR4^1013^ rafts (Fig 4, panels B-C). Consistent with these results, p53 levels were significantly lower in CXCR4^1013^-rafts than in CXCR4^wt^ rafts (Fig 4, panel D). These results demonstrate that CXCR4^1013^ plays an important role in driving the HPV18 viral life cycle toward carcinogenesis, notably by increasing the levels of the HPV oncoproteins.

### Inhibition of the ATM DNA damage pathway in CXCR4^1013^-expressing rafts

**Fig 5:**
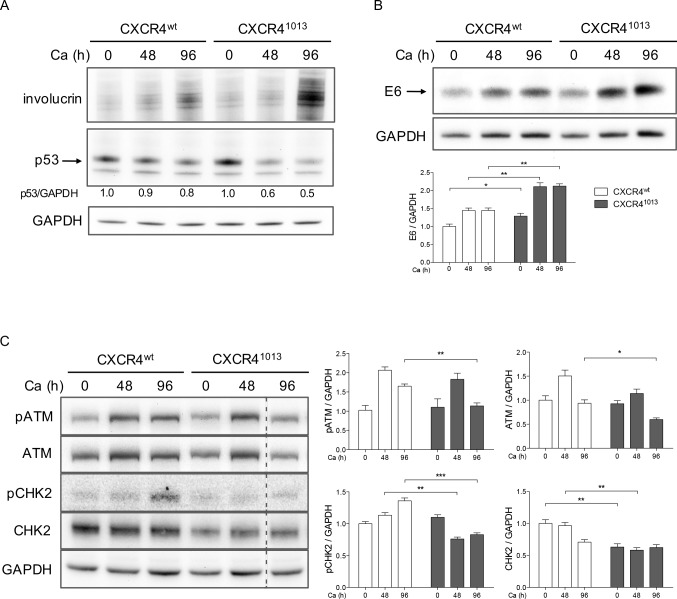
Inhibition of the ATM DNA damage pathway in CXCR4^1013^-expressing rafts. Western blots and densitometric analyses showing relative levels of involucrin and p53 (A), HPV18-E6 (B), pATM, ATM, pCHK2 and CHK2 (C) in HPV18-positive CXCR4^wt^ and CXCR4^1013^ NIKS cells differentiated in high calcium media (Ca) for indicated times. Levels of target proteins were normalized to GAPDH protein levels and arbitrarily set at 1 for undifferentiated (Ca 0 h) CXCR4^wt^ NIKS cells. Values are means ± SEM. *p < 0.05, **p < 0.01, and ***p < 0.001.

HPV, like other viruses associated with human cancers, was recently shown to hijack DNA damage response pathways responsible for maintaining genomic integrity. Proteins involved in these pathways include the ataxia telangiectasia mutated (ATM) and Rad3-related protein kinases, as well as p53 and the CHK1 and CHK2 kinases, which are involved in downstream checkpoint pathways [17]. Activation of the ATM/CHK pathway in the course of HPV infection provides a suitable environment for viral replication in differentiated cells [37]. The mechanisms of this activation, which may be part of the HPV replication process itself, are not completely understood but may involve the E1, E2, and E7 proteins [38]. To investigate whether the ATM/CHK pathway can be differentially modified in a CXCR4^1013^-expressing background as a result of altered viral replication or higher E7 expression levels, we analyzed the expression of the ATM and CHK proteins in keratinocytes differentiated in high-calcium medium. Progression of normal differentiation was indicated by increases in involucrin protein abundance over time in CXCR4^1013^- and CXCR4^wt^-expressing NIKS cells cultured in high-calcium medium (Fig 5, panel A). In differentiated HPV18-positive NIKS cells, E6 protein levels were higher in cells expressing CXCR4^1013^ than in those expressing CXCR4^wt^ (Fig 5, panel B). These results confirmed and extended our findings in raft cultures (Fig 3). Confirming our results in raft cultures (Fig 4), p53 protein levels at 96 h were lower in CXCR4^1013^-expressing cells than in CXCR4^wt^-expressing cells (Fig 5, panel A), which was likely related to the higher levels of E6 protein in CXCR4^1013^-expressing cells. The expression patterns of ATM and its activated phosphorylated form (pATM) in control cultures (Fig 5, panel C, CXCR4^wt^-cells) were concordant with previous report [37]. After 96 h of culture, pATM levels were lower in CXCR4^1013^-expressing cells than in CXCR4^wt^-expressing cells (Fig 5, panel C). Accordingly, the levels of the activated phosphorylated form of CHK2 (pCHK2) kinase were significantly lower in keratinocytes expressing CXCR4^1013^ than in those expressing CXCR4^wt^ at 96 h but also at 48 h (Fig 5, panel C). This correlates with the tendency of CXCR4^1013^-expressing cells to have lower levels of activated ATM. Altogether these results suggest that the presence of CXCR4^1013^ might lead to suboptimal activation of the ATM pathway and a reduced ability to support HPV replication.

### Normalizing β-arrestin-dependent signaling partially restored the productive HPV life cycle

**Fig 6:**
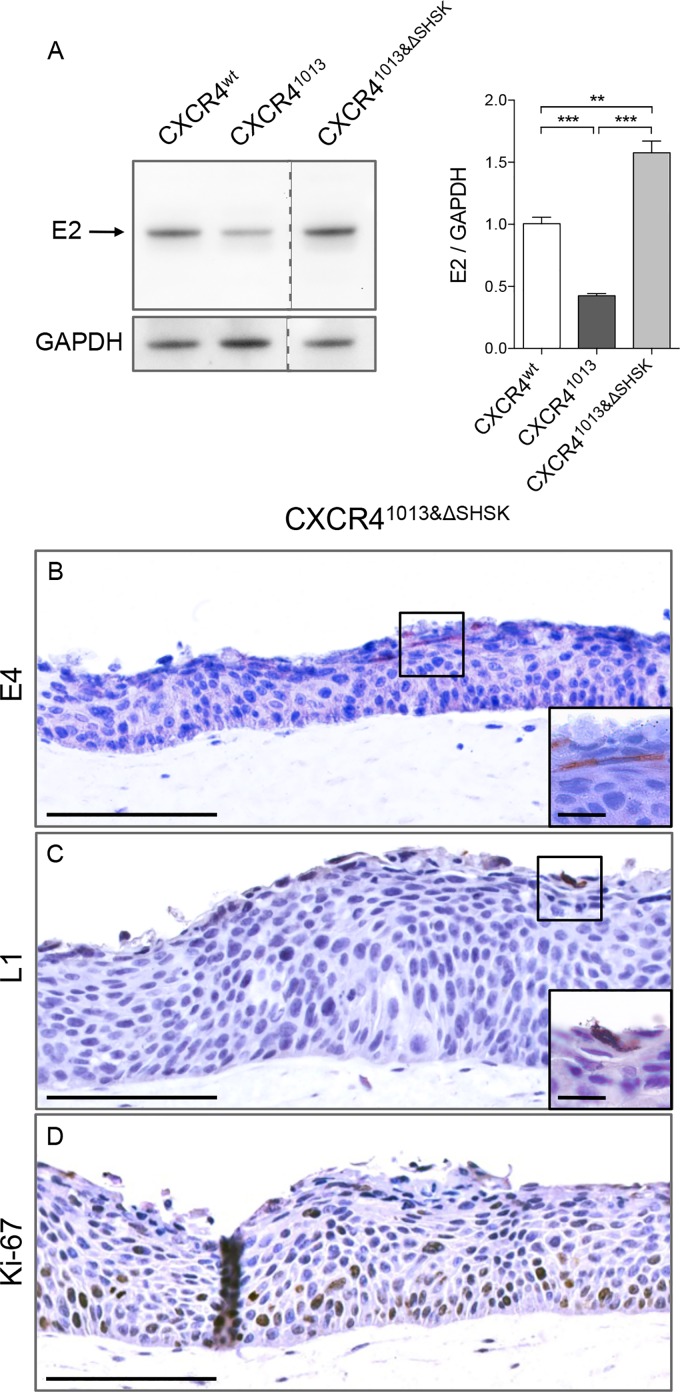
Normalizing β-arrestin-dependent signaling of CXCR4^1013^ partially restores the productive HPV life cycle. (A) Western blots (left) and densitometric analyses (right) showing relative levels of HPV18-E2 protein in HPV18-positive CXCR4^wt^, CXCR4^1013^, and CXCR4^1013&ΔSHSK^ raft cultures. E2 protein levels were normalized to GAPDH protein levels and arbitrarily set at 1 for CXCR4^wt^ rafts (staining controls are shown in S5 Fig). Values are means ± SEM. **p < 0.01 and ***p < 0.001. HPV18-positive CXCR4^1013&ΔSHSK^ raft sections stained for HPV18-E4 (B), HPV18-L1 (C), and Ki-67 (D) expression by immunohistochemical staining. Images are representative of three independent experiments. Scale bars = 100 μm, inset scale bar = 10 μm.

The suboptimal HPV replication environment in keratinocytes expressing CXCR4^1013^ prompted us to examine the consequences of normalizing the CXCR4^1013^-enhanced signaling. One of the key pathways accounting for the CXCR4^1013^ gain-of-function is the enhanced β-arrestin-mediated signaling, which is dependent upon the third intracellular loop (SHSK motif) of CXCR4 [39], which is also required for mobilization of intracellular calcium and G-protein-independent stimulation of JAK2/STAT3 in response to CXCL12 [40]. Therefore, we expressed CXCR4^1013^ lacking the SHSK motif, CXCR4^1013&ΔSHSK^ in keratinocyte raft cultures (Fig 6). In leukocytes, CXCR4^1013&ΔSHSK^ exhibited normal β-arrestin–mediated signaling and CXCL12-induced chemotaxis [39]. In raft cultures expressing CXCR4^1013&ΔSHSK^, E2 protein levels were significantly higher than in CXCR4^1013^-rafts (Fig 6, panel A). E4 and L1 proteins were detected in the upper epithelial layers of the CXCR4^1013&ΔSHSK^-rafts (Fig 6, panels B-C). Proliferating cells expressing the Ki-67 antigen remained confined to basal and suprabasal layers (Fig 6, panel D), as in CXCR4^wt^ raft (Fig 4, panel A). Thus, CXCR4^1013&ΔSHSK^, with normal β-arrestin-mediated signaling, lacks the ability to impair virus production, further supporting the capacity of CXCR4 to tune the viral life cycle through its downstream signaling pathways.

### Blockade of CXCR4 function diminishes oncogene expression

**Fig 7:**
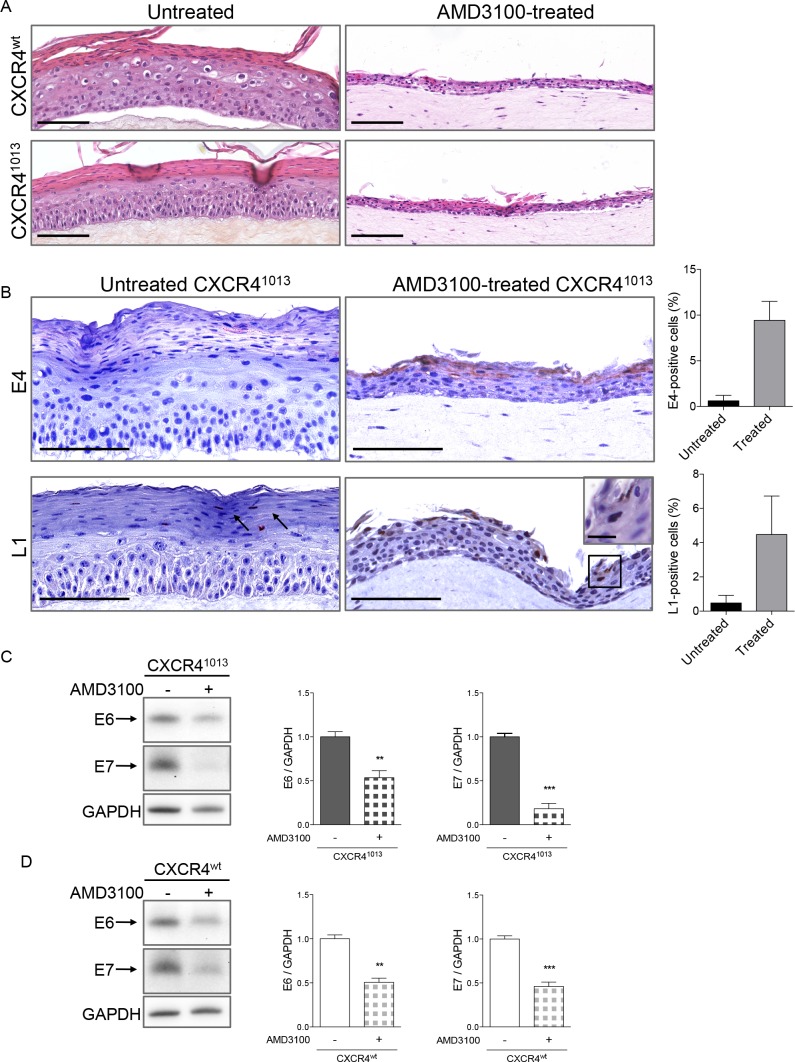
Blocking CXCR4^1013^ activity by the selective antagonist AMD3100 restores the productive HPV life cycle. (A) Sections of HPV18-positive CXCR4^wt^ and CXCR4^1013^ raft cultures treated (AMD3100-treated) or not (untreated) with AMD3100 and stained with hematoxylin and eosin. (B) Representative HPV18-positive CXCR4^1013^ raft cultures sections treated or not with AMD3100 and stained for HPV18-E4 (upper panel) or HPV18-L1 (lower panel). Images are representative of three independent experiments. Scale bars = 100 μm, inset scale bar = 10 μm. Quantifications of E4- and L1-positive cells in untreated and AMD3100-treated (treated) rafts from histological analyses are expressed as the percentage of positive cells out of the total number of epithelial cells. Values are means ± SEM. (C-D) Western blots (left) and densitometric analyses (right) showing relative levels of HPV18-E6 and HPV18-E7 proteins in HPV18-positive CXCR4^1013^ (C) and CXCR4^wt^ (D) raft cultures treated or not with AMD3100. E6 and E7 protein levels were normalized to GAPDH and arbitrarily set at 1 for untreated raft cultures. Values are means ± SEM. **p < 0.01 and ***p < 0.001.

**Fig 8:**
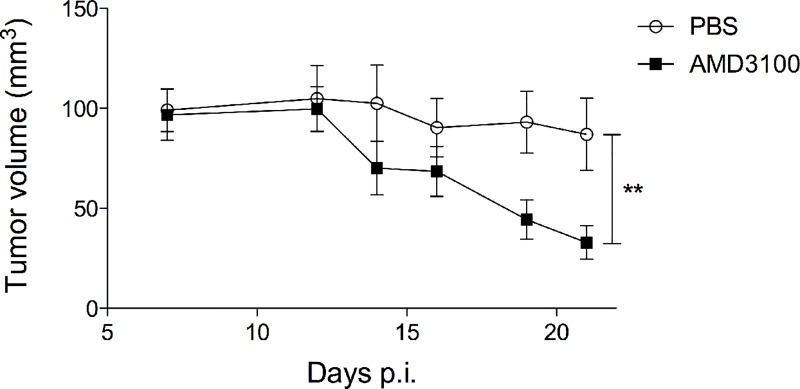
AMD3100 treatment reduces the size of the tumors induced by injection of HPV18-positive keratinocytes expressing CXCR41013 in nude mice. Tumor growth curve of the average tumor volume (mm3 ± SEM) in each group (n = 6 for AMD3100-treated mice and n = 7 for PBS-control mice) as a function of time. Representative results from one of two independent experiments are shown. **p < 0.01 (two-way ANOVA).

To further assess the role of CXCR4 activity in the HPV life cycle, CXCR4^wt^- and CXCR4^1013^-rafts were treated with AMD3100, a selective and competitive antagonist of CXCR4 [41] that efficiently blocks CXCR4^1013^ function [28]. The architecture and viral expression patterns of raft cultures treated with AMD3100 were compared to control ones (untreated rafts) by immunohistochemistry and western blot analyses (Fig 7). The stratified epithelium in CXCR4^wt^ and CXCR4^1013^-rafts treated with AMD3100 was thinner than in untreated rafts (Fig 7, panel A) but keratinocytes differentiation was apparently not affected given the normal expression pattern for keratin 10 (S9 Fig panel A). E4 and L1 proteins were readily detected in the upper layers of CXCR4^1013^-rafts treated with AMD3100 (Fig 7, panel B) as in CXCR4^wt^-rafts treated with AMD3100 (S9 Fig panel B), while sparsely detected (L1) or undetectable (E4) in untreated CXCR4^1013^-raft cultures (Fig 7, panel B). In contrast, AMD3100 treatment reduced expression of the E6 and E7 oncoproteins in CXCR4^1013^- and CXCR4^wt^-rafts (Fig 7, panel C and D, respectively). Thus blocking CXCR4^1013^-dependent signaling allows virus production, while reducing oncogene-expression and–driven neoplastic-like changes. Moreover, we found that tumors produced by injecting CXCR4^1013^-expressing human keratinocytes immortalized by HPV18 [29] into nude mice were significantly smaller in AMD3100-treated mice than in untreated mice (Fig 8). Collectively, these results indicate that CXCL12/CXCR4 signaling impacts the balance between HPV replication and HPV-driven carcinogenesis.

## Discussion

Considering the role of the CXCL12/CXCR4-signaling in the migration and survival of HPV18-infected keratinocytes, we investigated the importance of this axis in the productive HPV life cycle in which the timely and coordinated expression of different viral genes occurs as infected cells move toward the epithelial surface where virions mature. In view of the consequences of axis deregulation in HPV-induced cell transformation via the CXCR4^1013^ gain-of-function mutant, we investigated the impact of CXCR4^1013^ expression on the productive HPV life cycle in an organotypic model of human epidermis.

Our results demonstrate that unregulated CXCR4^1013^ function fosters a keratinocyte environment that restrains the productive HPV18 life cycle in contrast to CXCR4^wt^-expressing rafts that efficiently reproduce the complete HPV life cycle including the production of infectious virions. These findings in CXCR4^1013^-rafts correlate with deregulated keratinocyte proliferation and the stabilization of the E6 and E7 oncoproteins together with dramatic decreases in production of the late viral proteins involved in HPV virion assembly. These results are especially relevant because CXCL12 is expressed in keratinocytes from HPV-infected raft cultures (S10 Fig). This extends previous studies detecting CXCL12 expression in epidermal keratinocytes from HPV-induced lesions but not from other skin pathologies and normal skin [28, 42]. Thus collectively, our results support the concept that the CXCL12/CXCR4 pathway controls the HPV productive life cycle, likely reflecting the normal function of this pathway as a regulator of keratinocyte proliferation and survival. When deregulated, as in the WHIM syndrome, the CXCL12/CXCR4 pathway can trigger HPV-induced transformation as a result of elevated levels of oncoprotein-induced signaling and down-regulation of DNA damage response pathways associated with cell hyperproliferation.

On the one hand, the severe pathogenesis in WHIM patients manifests as intractable genital warts that often develop into severe dysplasia and carcinoma [43, 44]. This pathogenesis is generally due to mucosal high-risk HPV types for which we can suggest that expression of CXCR4^1013^ might enhance transforming capacity, partly through the elevation of E6/E7 proteins expression that drive proliferation of the infected cells, although this remains to be investigated directly in patients-derived cells. In some cases, patients’ dysplasia was found to be associated with low-risk HPV types, such as HPV6, which display potential oncogenicity [45]. On the other hand, the profusion and persistency of cutaneous warts is another major clinical manifestation of the WHIM-associated HPV pathogenesis. In this regard, it can be postulated a role for CXCR4^1013^ in driving the initial proliferation of the infected cells, thus allowing expression and replication of cutaneous low-risk HPV types, which do not normally stimulate proliferation. Our study is providing the rational for future mechanistic investigations of the productive life cycle of cutaneous low-risk HPV, which remains underappreciated due to the lack of robust in vitro models.

We have found that CXCR4^1013^ expression in keratinocyte raft cultures is associated with the stabilization of the E6 and E7 oncoproteins. This was not associated with an integration of the HPV genome further supporting that disruption of the E2 ORF is not the only mechanism of suppressing E2 and increasing E6 and E7 expression as previously reported in HPV16-induced carcinogenic progression [46]. Such modulations of oncoproteins expression might involve the ubiquitin-proteasome system, which is involved in the degradation of E6 produced by the high-risk types HPV18 and HPV16 [47]. Additionally, the capacity of E6 to interact with certain PDZ domain proteins and phosphoserine-binding proteins involved in cell signaling pathways is a mechanism for regulating E6 stability that is common to diverse high-risk HPV types [48–50]. Beside PDZ domain proteins, chemokine receptors including CXCR4 can interact with chaperone proteins, some being recently found to increase the steady-state levels and half-life of E6 and E7 oncogenes [51, 52]. As the interaction of E6 with either of these protein families depends on its phospho-regulation by various kinases (e.g. protein kinase A or B) and determines the fate of E6, different environmental conditions might have a significant impact on the likelihood of HPV infection progressing toward malignancy [53]. We propose that changes in cell signaling pathways in the context of CXCR4^1013^ expression, and notably in the downstream kinases activated by CXCL12-CXCR4 signaling may differentially affect the stability of HPV18 E6. Some kinases, as well as the rate of ubiquitination, can also control the steady-state level of E7 by interfering with proteasome-dependent degradation, but these processes have been studied in the context of only a few HPV types (HPV16 and HPV6) [54, 55].

Increased production of E6 and E7 proteins in the context of CXCR4^1013^ expression makes rafts prone to drive the viral lifecycle toward carcinogenesis as demonstrated by the altered levels of keratinocyte proliferation and apoptosis and by a disturbance in the ordered expression of viral gene products that normally leads to virus replication and production. Whether the fact that rafts derive from the spontaneously immortalized human NIKS keratinocytes might contribute to this process is not known and is awaiting the setting of rafts from primary human keratinocytes for HPV life cycle modeling.

Previous studies have clearly suggested that an increase in high-risk HPV protein levels can drive a more severe neoplastic phenotype [56]. Among these viral proteins, we observed a dramatic decrease in the level of E2 that might be related to enhanced degradation by the ubiquitin-proteasome system, which controls viral protein stability and was proposed to operate in cycling cells [57]. Aberrant cell cycle progression in CXCR4^1013^-rafts might thus increase E2 degradation and diminish its activity in the late phases of the viral life cycle. Conversely, normalizing arrestin-dependent signaling downstream of CXCR4^1013^ (CXCR4^1013&ΔSHSK^) might stabilize E2 protein thus partially restoring the productive HPV life cycle. This shift toward viral production was revealed by the enhanced production of L1 and E4, which are primarily involved in genome packaging and virus release [1, 58, 59]. The stabilization of E2 in CXCR4^1013&ΔSHSK^-rafts might also be accounted for the physical and functional interaction of E2 and E4 as the level of each protein is increased by the presence of the other [60].

Additionally, and consistent with the recently reported essential role of DNA damage responses in the viral replication [37], ATM/CHK pathway activation was found to be significantly lower in CXCR4^1013^-rafts. The contribution of ATM/CHK pathway activation to viral replication has begun to be deciphered but its function in HPV-induced cancer development remains unclear, especially because E7 and E6 have important roles in promoting this activation [38, 61]. Decreased activation of the ATM/CHK proteins in CXCR4^1013^-rafts, in spite of increased expression of E6 and E7, may appear paradoxical. However, such deregulation might include the JAK/STAT pathway, which is induced downstream of CXCR4 in a G protein-independent manner [62] and was shown to activate the ATM-dependent DNA damage responses [38]. Although the biological role of DNA damage responses in HPV-induced malignancy is still uncertain, decreasing the production or activation of ATM/CHK proteins would likely lead to the accumulation of DNA damage in CXCR4^1013^-rafts.

In cervical disease, it is thought that the levels of E6 and E7 rise with cervical intraepithelial neoplasia (CIN) severity. Changes in gene expression underlie the neoplastic progression, with CIN1 lesions supporting the complete HPV life cycle in contrast to CIN3 lesions that are considered to be high-grade precancerous. We provide evidence that blocking CXCL12/CXCR4-dependent signaling in the course of raft culture differentiation allowed the productive HPV life cycle to proceed at the expense of the HPV-induced carcinogenesis in CXCR4^1013^-rafts. These results provide molecular clues to the potential therapeutic effect of AMD3100 treatment for skin warts when combined with imiquimod [63] but also for HPV-induced carcinogenesis in a mouse preclinical model [64] and here in nude mice after injection of human keratinocytes immortalized by HPV18 and expressing CXCR4^1013^. Whereas we think that the interplay between the HPV life cycle and CXCL12/CXCR4 in keratinocytes is the direct mediator element, the beneficial effect of AMD3100 might also be related to other components that are controlled by this signaling axis (e.g. resident and infiltrating immune cells in the skin, stem cell recruitment, or endothelial cell responses). The CXCL12/CXCR4 pair is indeed involved in the increased survival and/or proliferation of cancer cells from various types including virus-related cancers, as well as in the promotion of tumor metastasis and angiogenesis linked to tumor progression [65–69].

In light of the HPV-pathogenesis associated with the WHIM syndrome, it can be extrapolated that dysfunction of CXCR4 might be acquired from genetic errors accumulated during the multistep process of HPV-induced neoplasia, as demonstrated by the somatic WHIM-like *CXCR4* mutations reported in Waldenström macroglobulinemia [70] or the anomalies in the effectors of the CXCL12/CXCR4-signaling pathway reported in patients with GATA2-deficiency [71–73]. Clues to the additional effectors of this potentially pathogenic process arise from the abnormal expression of CXCL12 in HPV-lesions in the general population of HPV infected individuals [28] and from the presence of CXCL12 (our data) among the panel of expressed genes unique to the discrete population of cuboidal cells located in the cervical squamocolumnar junction, which have been linked to HPV-related cervical intraepithelial neoplasia and cancers [5]. Consequently, the CXCL12/CXCR4 signaling pathway appears to be an important host factor in HPV-induced pathogenesis.

## Materials and Methods

### Routine cell culture

NIKS cells, near-diploid spontaneously immortalized human keratinocytes [31] (kindly provided by Dr Paul F. Lambert), were maintained at subconfluence on mitomycin C-treated 3T3-J2 feeder cells (kindly provided by Dr Paul F. Lambert) in F medium with all supplements as previously described [30]. Human foreskin fibroblasts (kindly provided by Dr Paul F. Lambert) were cultured in Ham’s F12 medium containing 10% fetal bovine serum and 1% penicillin-streptomycin, before use in raft cultures.

### Generation of HPV18-positive NIKS cells expressing CXCR4-derived receptors

NIKS cells expressing CXCR4^wt^, CXCR4^1013^ or CXCR4^1013&ΔSHSK^ were obtained with a lentivirus-mediated strategy as previously described [29, 39]. Expression of similar levels of each receptor in the different cell populations was checked by flow cytometry (S1 Fig, panel B). Recircularized HPV18 DNA was prepared as previously described [30]. The different NIKS cell populations (2.5 x 10^6^ cells plated the day before the transfection) were cotransfected with 2 μg of recircularized HPV18 DNA and 0.5 μg of the blasticidin-resistance plasmid pcDNA6 using Effectene Transfection Reagent (QIAGEN). Blasticidin selection (7 μg/mL) was performed for 6 days.

### Organotypic raft cultures

HPV18-positive NIKS cells expressing CXCR4^wt^, CXCR4^1013^, or CXCR4^1013&ΔSHSK^ were grown in raft cultures to induce the three-dimensional architecture of the stratified epithelium, as described previously [30]. Briefly, 1.5 x 10^6^ NIKS cells were seeded onto a dermal equivalent composed of rat-tail collagen type 1 containing 1 x 10^6^ human foreskin fibroblasts. Raft cultures were lifted onto transwell inserts submerged in deep well plates containing keratinocyte plating medium and cultured for 4 days. The transwell inserts were then raised by placing four cotton pads underneath them, thereby exposing the epithelial cells to the air-liquid interface. Raft cultures were fed by diffusion from below with cornification medium and were allowed to stratify for 14 days. When specified, AMD3100 (A5602, Sigma-Aldrich) was added to the cornification medium at a concentration of 20 μg/mL from day 4 to the end of the experiment. Rafts were then removed from transwell inserts and either fixed in formalin and embedded in paraffin for histological analyses, or frozen at −80°C for quantitative real-time PCR and western blot analyses.

### Calcium-induced differentiation

To induce differentiation in medium containing 1.5 mM CaCl_2_ (high calcium), HPV18-positive NIKS cells expressing either CXCR4^wt^ or CXCR4^1013^ were cultured in absence of 3T3-J2 feeders in F medium for 24 h and then switched to F medium (without growth supplements) containing 1.5 mM CaCl_2_. Cells were then harvested at 0, 48, and 96 hours for protein extraction.

### Western blot analysis

NIKS cells from monolayer cultures or rafts cultures were resuspended in protein lysis buffer (1% Triton X-100, 10 mM Tris-HCl pH 7.4, supplemented with Protease and Phosphatase Inhibitor Tablets (Pierce)). Protein concentration was measured with the BCA Protein Assay Kit (Pierce) according to the manufacturer’s protocol. Equivalent amounts of protein were separated on a SDS-polyacrylamide gel and transferred to a PVDF membrane. Primary antibodies were as follows: anti-HPV18-E6 (kindly provided by Arbor Vita Corporation), anti-HPV18-E7 (sc-1590, Santa Cruz), anti-HPV18-E2 (kindly provided by Dr. F. Thierry), anti-p53 (sc-126, Santa Cruz), anti-involucrin (I9018, Sigma-Aldrich), anti-GAPDH (14–9523, eBioscience); anti-pATM (Ser1981; #13050), anti-ATM (#2873), and anti-pCHK2 (Thr68) and anti-CHK2 (#2661 and #3440, respectively, Cell Signaling). Membranes were incubated with the appropriate secondary antibodies conjugated to HRP (GE Healthcare). Proteins were detected using the Immobilon Western Chemiluminescent HRP kit (Millipore).

### Histological analysis

Raft paraffin sections (5-μm thick) were stained with hematoxylin and eosin (HE), and with safran (HES) where indicated. Immunohistochemistry was performed on paraffin sections using primary antibodies for keratin 10 (MA1-35540, Thermo Scientific), filaggrin (VP-F706, Vector laboratories), HPV18-E2 (provided by Dr. F. Thierry), HPV18-E4 (provided by Dr. J. Doorbar), HPV18-L1, p16 (sc-56330, Santa Cruz), Ki-67 (18-0191Z, AbCys), and CXCL12 (K15C clone, MABC184, EMD Millipore). Bound antibodies were detected using the LSAB+/HRP kit (K0679, Dako) or the AEC+ High Sensitivity Substrate Chromogen Ready-to-Use System (K3461, Dako). For immunofluorescence, staining with the primary antibody for MCM2 (ab31159, Abcam) was followed by staining with a goat anti-rabbit Alexa Fluor 596 (Invitrogen). Tissues were counterstained with DAPI. For the TUNEL assay to detect apoptotic cells, we used the In Situ Cell Death Detection Kit, Fluorescein (11684795910, Roche Diagnostics) according to the manufacturer’s instructions. In situ hybridization was performed with the wide spectrum HPV biotinylated DNA probe sets able to detect 11 types of anogenital HPV (In Situ Hybridization Detection System, K0601, Dako).

### Image analysis

Where indicated, slides were scanned by the digital slide scanner NanoZoomer 2.0-RS (Hamamatsu) allowing an overall view of the samples. Images were digitally captured from the scanned slides using the NDP.view2 software (Hamamatsu). All quantifications from the histological analyses were performed by counting 10 different fields on the scanned slides. Other slides were analyzed using a Leica DMLA microscope, in particular to visualize images at 100X magnification, and images were captured with a Leica DFC450 C digital microscope camera. Immunochemical stainings were interpreted simultaneously and independently by at least two investigators (FM, LC, or FG).

### Nude mice xenografts and AMD3100 treatment

HK-HPV18-CXCR4^1013^ tumors were established in nude mice as previously described [29]. Briefly, athymic female nude nu/nu 5-week-old mice (Harlan Laboratories) were injected subcutaneously with 2 x 10^7^ HK-HPV18 cells expressing T7-GFP-tagged CXCR4^1013^ in the right flank (six to seven mice per group). Mice were treated with 5 mg/kg of AMD3100 (intraperitoneal administration) on days 8, 13, 16, and 20 after tumor cell injection. Tumor volumes (V) were calculated as V = π/6 x (length x width^2^).

### Ethics Statement

All experimental procedures were conducted in our animal facility (agreement n° B 92-023-01) in accordance with the European Union’s legislation and the relevant national legislation, namely the French “Décret no 2013–118, 1er février 2013, Ministère de l’Agriculture, de l’Agroalimentaire et de la Forêt” regarding the use of laboratory animals and were approved by the Committee on the Ethics of Animal Experiments (Comité d'Ethique en Expérimentation Animale Capsud or CEEA-26) under the authorization 2014_039 #2521.

### Statistical analysis

Student’s t test was used to compare the significance between specified groups. All analyses were performed with GraphPad Prism software.

## Supporting Information

S1 Fig Expression of CXCR4 receptors in HPV18-positive keratinocyte NIKS cell lines in monolayer and raft cultures. (A) Cell surface expression of CXCR4 in CHO cells transfected with 1 and 2.5 μg of a vector encoding for the human CXCR4 receptor was investigated by flow cytometry using the 12G5 antibody, which specifically recognizes the human form of CXCR4 and not the endogenous Chinese hamster CXCR4 receptor. (B) Cell surface expression of CXCR4 in HPV18-positive NIKS cells, non-transduced (i.e. endogenous (Endo.) CXCR4) as compared to cells transduced with lentiviral vectors expressing CXCR4^wt^ or CXCR4^1013^ (left graph) or to uninfected NIKS cells (right graph). Cell surface expression of CXCR4, investigated by flow cytometry using the 12G5 antibody, is represented as mean fluorescence intensity (MFI) ± SEM (n = 3). (C) HPV18-positive NIKS cells non-transduced (i.e. endogenous CXCR4) or transduced with lentiviral vectors expressing CXCR4^wt^ or CXCR4^1013^ were investigated for CXCR4 transcripts levels. Transcripts were expressed as relative levels normalized to GAPDH transcripts levels (mean ± SEM, n = 3). (D) Detection of CXCR4 expression by immunofluorescence in HPV18-positive raft cultures sections (i.e. endogenous CXCR4) and in HPV18-positive CXCR4^wt^ and CXCR4^1013^ raft culture sections. Images are representative of three independent experiments. Scale bars = 100 μm, inset scale bars = 20 μm.
(TIF)

S2 Fig Viral DNA and transcripts in CXCR4^wt^ and CXCR4^1013^ NIKS cells in monolayer and in raft cultures. (A) HPV18-positive NIKS cells non-transduced (i.e. endogenous CXCR4) or transduced with lentiviral vectors expressing CXCR4^wt^ or CXCR4^1013^ were investigated for HPV18 DNA copy numbers before (i.e. HPV18-infected NIKS) and after being differentiated into 3D cultures (i.e. HPV18-infected rafts). Uninfected rafts were also integrated as negative control. HPV18 DNA copy numbers are expressed as the ratio to *GAPDH* gene copy numbers (mean ± SEM, n = 3). (B) HPV18-E6/E7 and HPV18-E2 transcripts levels in HPV18-positive CXCR4^wt^ and CXCR4^1013^ NIKS cells cultured in monolayers before being differentiated into raft cultures (see S6 Fig). Transcripts were expressed as relative levels normalized to GAPDH transcripts levels (mean ± SEM, n = 3).
(TIF)

S3 Fig Architecture of HPV18-positive raft cultures developed from keratinocytes expressing endogenous CXCR4 only. Representative section of HPV18-positive raft cultures stained with hematoxylin and eosin (HE; upper panel) and for HPV18-E4 protein (lower panel). Images are representative of three independent experiments. Scale bars = 100 μm.
(TIF)

S4 Fig Analysis of infectious virus progeny. HaCat cells were infected with a 1:20 or 1:100 dilution of viral stocks harvested from either HPV18-positive CXCR4^wt^ or CXCR4^1013^ raft cultures. Shown is a 2% agarose gel of nested RT-PCR-amplified β-actin and HPV18 E1^E4. Lane 1, CXCR4^wt^ HPV18 at 1:20. Lane 2, CXCR4^wt^ HPV18 at 1:100. Lane 3, CXCR4^1013^ HPV18 at 1:20. Lane 4, CXCR4^1013^ HPV18 at 1:100. Lane 5, negative control (no virus). β-actin and HPV18 E1^E4 sequences were confirmed by sequencing and positions are indicated in the right and molecular size markers are indicated in the left.
(TIF)

S5 Fig Control experiments for E2, E6 and E7 antibodies specificity. Western blots showing detection of HPV18-E2, HPV18-E6 and HPV18-E7 proteins in uninfected (negative control for the detection of HPV18 proteins) versus HPV18-infected conditions (rafts or NIKS cells). Proteins were extracted from raft cultures (left panel) or NIKS cells (central and right panels). GAPDH detection and size markers are also shown.
(TIF)

S6 Fig Virus transcription and integration in HPV18-positive raft cultures and LCR activity in NIKS cells. HPV18-positive CXCR4^wt^ and CXCR4^1013^ raft cultures were investigated (A) for HPV18-E6/E7 and HPV18-E2 transcripts levels (transcripts were expressed as relative levels normalized to GAPDH transcripts levels (mean ± SEM, n = 3)), and (B) for HPV integration using the APOT assay. Shown is a 1.2% agarose gel of nested RT-PCR-amplified HPV E7. Lane 1, negative control (HaCat cells); lanes 2 and 3, positive controls (Human keratinocytes and HeLa cells, respectively, containing integrated HPV18 genome); lanes 4 to 6, HPV18-infected NIKS, CXCR4^wt^ NIKS and CXCR4^1013^ NIKS, respectively; lanes 7 and 10, HPV18 infected NIKS-derived rafts; lanes 8 and 11, CXCR4^wt^-rafts; Lanes 9 and 12, CXCR4^1013^-rafts. Molecular size markers are indicated in the right and positive controls in lanes 2 and 3 were confirmed by sequencing. (C) Luciferase reporter assays was used to investigate the intrinsic promoter activity of the HPV18 LCR in NIKS cells transduced for expression of CXCR4^wt^ or CXCR4^1013^, and transiently transfected with the LCR-HPV18-luciferase vector. Luciferase ratio represents the fold increase of luciferase signal over the luciferase activity in cells transfected with the control pClucF plasmid (mean ± SEM, n = 3).
(TIF)

S7 Fig CXCR4^1013^-mediated transforming properties involved stabilization of the E6 and E7 HPV oncoproteins in NIKS cells. Western blots (upper panels) and densitometric analyses (lower panels) showing relative levels of HPV18-E6 and HPV18-E7 in HPV18-positive CXCR4^wt^ and CXCR4^1013^ NIKS cells differentiated in high-calcium media for 96 h (A) or undifferentiated (B). NIKS cells were treated with cycloheximide (50 μg/mL) for the indicated times. Densitometric analyses represent E6 and E7 protein levels normalized to GAPDH protein levels and expressed as percent of the initial levels at time 0 set at 100%. (A) E6 protein levels were 0.19 +/- 0.01; 0.14 +/- 0.01; 0.086 +/- 0.008 and 0.25 +/- 0.01; 0.167 +/- 0.01; 0.168 +/- 0.01 (at 0, 1 and 2 h post cycloheximide for CXCR4^wt^ and CXCR4^1013^, respectively). E7 protein levels were 2.0 +/- 0.12; 2.13 +/- 0.10; 1.34 +/- 0.16; 0.44 +/- 0.01 and 1.18 +/- 0.029; 1.34 +/- 0.019; 1.23 +/- 0.023; 0.79 +/- 0.03 (0, 1, 2 and 4 h post cycloheximide for CXCR4^wt^ and CXCR4^1013^, respectively). (B) E6 protein levels were 0.56 +/- 0.10; 0.48 +/- 0.10; 0.41 +/- 0.10; 0.30 +/- 0.09 and 0.76 +/- 0.09; 0.71 +/- 0.11; 0.64 +/- 0.10; 0.43 +/- 0.11 (0, 1, 2 and 4 h post cycloheximide for CXCR4^wt^ and CXCR4^1013^, respectively). E7 protein levels were 0.3 +/- 0.08; 0.24 +/- 0.07; 0.19 +/- 0.07; 0.17 +/- 0.06 and 0.42 +/- 0.09; 0.38 +/- 0.10; 0.32 +/- 0.09; 0.27 +/- 0.09 (0, 1, 2 and 4 h post cycloheximide for CXCR4^wt^ and CXCR4^1013^, respectively). Values are the mean ± SEM. **p < 0.01 and ***p < 0.001.
(TIF)

S8 Fig Architecture of HPV-negative raft cultures. Representative sections of HPV-negative (HPV-) CXCR4^wt^ and CXCR4^1013^ raft cultures stained with hematoxylin and eosin (HE; upper panel), or for Ki-67 or keratin 10 expression (middle and lower panel, respectively). Images are representative of three independent experiments. Scale bars = 100 μm.
(TIF)

S9 Fig Keratin 10 and HPV18-E4 expression in raft cultures treated with AMD3100. Representative sections of HPV18-positive CXCR4^wt^ and CXCR4^1013^ raft cultures treated (AMD3100-treated) or not (untreated) with AMD3100 were investigated (A) for keratin 10, and (B) for HPV18-E4 expression. Images are representative of three independent experiments. Scale bars = 100 μm (A) and as shown (B).
(TIF)

S10 Fig CXCL12 expression in epidermal keratinocytes from HPV18-positive raft cultures. Representative sections of HPV18-positive CXCR4^wt^ and CXCR4^1013^ raft cultures stained for CXCL12 protein. Immunohistochemistry was performed using primary antibody for CXCL12.
(TIF)

S1 Protocol HPV detection by quantitative real-time PCR analysisLuciferase assayAPOT assayVirus isolation and in vitro infectivity essayExpression of CXCR4 by flow cytometry, quantitative real-time PCR and immunofluorescence
(DOCX)

## References

[1] Doorbar J, Egawa N, Griffin H, Kranjec C, Murakami I. Human papillomavirus molecular biology and disease association. Rev Med Virol. 2015;25 Suppl 1:2–23. 25752814 10.1002/rmv.1822 PMC5024016

[2] Antonsson A, Forslund O, Ekberg H, Sterner G, Hansson BG. The ubiquity and impressive genomic diversity of human skin papillomaviruses suggest a commensalic nature of these viruses. Journal of virology. 2000;74(24):11636–41. 11090162 10.1128/jvi.74.24.11636-11641.2000 PMC112445

[3] Lecuit M, Eloit M. The human virome: new tools and concepts. Trends Microbiol. 2013;21(10):510–5. 10.1016/j.tim.2013.07.001 23906500 PMC7172527

[4] Foulongne V, Sauvage V, Hebert C, Dereure O, Cheval J, Gouilh MA, et al Human skin microbiota: high diversity of DNA viruses identified on the human skin by high throughput sequencing. PLoS One. 2012;7(6):e38499 10.1371/journal.pone.0038499 22723863 PMC3378559

[5] Herfs M, Yamamoto Y, Laury A, Wang X, Nucci MR, McLaughlin-Drubin ME, et al A discrete population of squamocolumnar junction cells implicated in the pathogenesis of cervical cancer. Proc Natl Acad Sci U S A. 2012;109(26):10516–21. 10.1073/pnas.1202684109 22689991 PMC3387104

[6] Mirkovic J, Howitt BE, Roncarati P, Demoulin S, Suarez-Carmona M, Hubert P, et al Carcinogenic HPV infection in the cervical squamo-columnar junction. J Pathol. 2015;236(3):265–71. 10.1002/path.4533 25782708 PMC4457596

[7] Durst M, Gissmann L, Ikenberg H, zur Hausen H. A papillomavirus DNA from a cervical carcinoma and its prevalence in cancer biopsy samples from different geographic regions. Proc Natl Acad Sci U S A. 1983;80(12):3812–5. 6304740 10.1073/pnas.80.12.3812 PMC394142

[8] zur Hausen H. Papillomaviruses in the causation of human cancers—a brief historical account. Virology. 2009;384(2):260–5. 10.1016/j.virol.2008.11.046 19135222

[9] McLaughlin-Drubin ME, Meyers J, Munger K. Cancer associated human papillomaviruses. Curr Opin Virol. 2012;2(4):459–66. 10.1016/j.coviro.2012.05.004 22658985 PMC3422426

[10] Karagas MR, Nelson HH, Sehr P, Waterboer T, Stukel TA, Andrew A, et al Human papillomavirus infection and incidence of squamous cell and basal cell carcinomas of the skin. J Natl Cancer Inst. 2006;98(6):389–95. 10.1093/jnci/djj092 16537831

[11] Mesri EA, Feitelson MA, Munger K. Human viral oncogenesis: a cancer hallmarks analysis. Cell Host Microbe. 2014;15(3):266–82. 10.1016/j.chom.2014.02.011 24629334 PMC3992243

[12] Vargas-Parada L. Pathology: Three questions. Nature. 2012;488(7413):S14–5. 10.1038/488S14a 22932435

[13] Gravitt PE. The known unknowns of HPV natural history. J Clin Invest. 2011;121(12):4593–9. 10.1172/JCI57149 22133884 PMC3225991

[14] Doorbar J. Molecular biology of human papillomavirus infection and cervical cancer. Clin Sci (Lond). 2006;110(5):525–41. 16597322 10.1042/CS20050369

[15] den Boon JA, Pyeon D, Wang SS, Horswill M, Schiffman M, Sherman M, et al Molecular transitions from papillomavirus infection to cervical precancer and cancer: Role of stromal estrogen receptor signaling. Proc Natl Acad Sci U S A. 2015;112(25):E3255–64. 10.1073/pnas.1509322112 26056290 PMC4485108

[16] Ojesina AI, Lichtenstein L, Freeman SS, Pedamallu CS, Imaz-Rosshandler I, Pugh TJ, et al Landscape of genomic alterations in cervical carcinomas. Nature. 2014;506(7488):371–5. 10.1038/nature12881 24390348 PMC4161954

[17] Weitzman MD, Weitzman JB. What's the damage? The impact of pathogens on pathways that maintain host genome integrity. Cell Host Microbe. 2014;15(3):283–94. 10.1016/j.chom.2014.02.010 24629335 PMC4501477

[18] Martens JE, Arends J, Van der Linden PJ, De Boer BA, Helmerhorst TJ. Cytokeratin 17 and p63 are markers of the HPV target cell, the cervical stem cell. Anticancer Res. 2004;24(2B):771–5. 15161025

[19] Yang EJ, Quick MC, Hanamornroongruang S, Lai K, Doyle LA, McKeon FD, et al Microanatomy of the cervical and anorectal squamocolumnar junctions: a proposed model for anatomical differences in HPV-related cancer risk. Mod Pathol. 2015;28(7):994–1000. 10.1038/modpathol.2015.54 25975286 PMC4490106

[20] Leiding JW, Holland SM. Warts and all: human papillomavirus in primary immunodeficiencies. J Allergy Clin Immunol. 2012;130(5):1030–48. 10.1016/j.jaci.2012.07.049 23036745 PMC3517887

[21] Hernandez PA, Gorlin RJ, Lukens JN, Taniuchi S, Bohinjec J, Francois F, et al Mutations in the chemokine receptor gene CXCR4 are associated with WHIM syndrome, a combined immunodeficiency disease. Nat Genet. 2003;34(1):70–4. 10.1038/ng1149 12692554

[22] Bachelerie F. CXCL12/CXCR4-axis dysfunctions: Markers of the rare immunodeficiency disorder WHIM syndrome. Dis Markers. 2010;29(3–4):189–98. 10.3233/DMA-2010-0736 21178277 PMC3835381

[23] Kawai T, Malech HL. WHIM syndrome: congenital immune deficiency disease. Curr Opin Hematol. 2009;16(1):20–6. 10.1097/MOH.0b013e32831ac557 19057201 PMC2673024

[24] Gulino AV. WHIM syndrome: a genetic disorder of leukocyte trafficking. Curr Opin Allergy Clin Immunol. 2003;3(6):443–50. 10.1097/01.all.0000104449.09202.d8 14612668

[25] McDermott DH, Gao JL, Liu Q, Siwicki M, Martens C, Jacobs P, et al Chromothriptic cure of WHIM syndrome. Cell. 2015;160(4):686–99. 10.1016/j.cell.2015.01.014 25662009 PMC4329071

[26] Tassone L, Moratto D, Vermi W, De Francesco M, Notarangelo LD, Porta F, et al Defect of plasmacytoid dendritic cells in warts, hypogammaglobulinemia, infections, myelokathexis (WHIM) syndrome patients. Blood. 2010;116(23):4870–3. 10.1182/blood-2010-03-272096 20736454

[27] Medler TR, Coussens LM. Duality of the immune response in cancer: lessons learned from skin. The Journal of investigative dermatology. 2014;134(e1):E23–8. 10.1038/skinbio.2014.5 25302470 PMC4950849

[28] Balabanian K, Lagane B, Pablos JL, Laurent L, Planchenault T, Verola O, et al WHIM syndromes with different genetic anomalies are accounted for by impaired CXCR4 desensitization to CXCL12. Blood. 2005;105(6):2449–57. 10.1182/blood-2004-06-2289 15536153

[29] Chow KY, Brotin E, Ben Khalifa Y, Carthagena L, Teissier S, Danckaert A, et al A pivotal role for CXCL12 signaling in HPV-mediated transformation of keratinocytes: clues to understanding HPV-pathogenesis in WHIM syndrome. Cell Host Microbe. 2010;8(6):523–33. 10.1016/j.chom.2010.11.006 21147466

[30] Lambert PF, Ozbun MA, Collins A, Holmgren S, Lee D, Nakahara T. Using an immortalized cell line to study the HPV life cycle in organotypic "raft" cultures. Methods Mol Med. 2005;119:141–55. 10.1385/1-59259-982-6:141 16353335

[31] Allen-Hoffmann BL, Schlosser SJ, Ivarie CA, Sattler CA, Meisner LF, O'Connor SL. Normal growth and differentiation in a spontaneously immortalized near-diploid human keratinocyte cell line, NIKS. The Journal of investigative dermatology. 2000;114(3):444–55. 10.1046/j.1523-1747.2000.00869.x 10692102

[32] McBride AA. The papillomavirus E2 proteins. Virology. 2013;445(1–2):57–79. 10.1016/j.virol.2013.06.006 23849793 PMC3783563

[33] Meyers C, Bromberg-White JL, Zhang J, Kaupas ME, Bryan JT, Lowe RS, et al Infectious virions produced from a human papillomavirus type 18/16 genomic DNA chimera. Journal of virology. 2002;76(10):4723–33. 10.1128/JVI.76.10.4723-4733.2002 11967289 PMC136126

[34] Griffin H, Soneji Y, Van Baars R, Arora R, Jenkins D, van de Sandt M, et al Stratification of HPV-induced cervical pathology using the virally encoded molecular marker E4 in combination with p16 or MCM. Mod Pathol. 2015;28(7):977–93. 10.1038/modpathol.2015.52 25953390 PMC4489599

[35] Moody CA, Fradet-Turcotte A, Archambault J, Laimins LA. Human papillomaviruses activate caspases upon epithelial differentiation to induce viral genome amplification. Proc Natl Acad Sci U S A. 2007;104(49):19541–6. 10.1073/pnas.0707947104 18048335 PMC2148325

[36] Zanier K, ould M'hamed ould Sidi A, Boulade-Ladame C, Rybin V, Chappelle A, Atkinson A, et al Solution structure analysis of the HPV16 E6 oncoprotein reveals a self-association mechanism required for E6-mediated degradation of p53. Structure. 2012;20(4):604–17. 10.1016/j.str.2012.02.001 22483108 PMC3325491

[37] Moody CA, Laimins LA. Human papillomaviruses activate the ATM DNA damage pathway for viral genome amplification upon differentiation. PLoS Pathog. 2009;5(10):e1000605 10.1371/journal.ppat.1000605 19798429 PMC2745661

[38] Hong S, Laimins LA. Regulation of the life cycle of HPVs by differentiation and the DNA damage response. Future Microbiol. 2013;8(12):1547–57. 10.2217/fmb.13.127 24266355 PMC3951404

[39] Lagane B, Chow KY, Balabanian K, Levoye A, Harriague J, Planchenault T, et al CXCR4 dimerization and beta-arrestin-mediated signaling account for the enhanced chemotaxis to CXCL12 in WHIM syndrome. Blood. 2008;112(1):34–44. 10.1182/blood-2007-07-102103 18436740

[40] Ahr B, Denizot M, Robert-Hebmann V, Brelot A, Biard-Piechaczyk M. Identification of the cytoplasmic domains of CXCR4 involved in Jak2 and STAT3 phosphorylation. J Biol Chem. 2005;280(8):6692–700. 10.1074/jbc.M408481200 15615703

[41] De Clercq E. The bicyclam AMD3100 story. Nat Rev Drug Discov. 2003;2(7):581–7. 10.1038/nrd1134 12815382

[42] Pablos JL, Amara A, Bouloc A, Santiago B, Caruz A, Galindo M, et al Stromal-cell derived factor is expressed by dendritic cells and endothelium in human skin. The American journal of pathology. 1999;155(5):1577–86. 10.1016/S0002-9440(10)65474-0 10550315 PMC1866989

[43] Dotta L, Tassone L, Badolato R. Clinical and genetic features of Warts, Hypogammaglobulinemia, Infections and Myelokathexis (WHIM) syndrome. Curr Mol Med. 2011;11(4):317–25. 21506920 10.2174/156652411795677963

[44] Beaussant Cohen S, Fenneteau O, Plouvier E, Rohrlich PS, Daltroff G, Plantier I, et al Description and outcome of a cohort of 8 patients with WHIM syndrome from the French Severe Chronic Neutropenia Registry. Orphanet J Rare Dis. 2012;7:71 10.1186/1750-1172-7-71 23009155 PMC3585856

[45] Tommasino M. The human papillomavirus family and its role in carcinogenesis. Semin Cancer Biol. 2014;26:13–21. 10.1016/j.semcancer.2013.11.002 24316445

[46] Xue Y, Lim D, Zhi L, He P, Abastado JP, Thierry F. Loss of HPV16 E2 Protein Expression Without Disruption of the E2 ORF Correlates with Carcinogenic Progression. Open Virol J. 2012;6:163–72. 10.2174/1874357901206010163 23341852 PMC3547325

[47] Lou Z, Wang S. E3 ubiquitin ligases and human papillomavirus-induced carcinogenesis. J Int Med Res. 2014;42(2):247–60. 10.1177/0300060513506655 24445694

[48] Nicolaides L, Davy C, Raj K, Kranjec C, Banks L, Doorbar J. Stabilization of HPV16 E6 protein by PDZ proteins, and potential implications for genome maintenance. Virology. 2011;414(2):137–45. 10.1016/j.virol.2011.03.017 21489588

[49] Boon SS, Banks L. High-risk human papillomavirus E6 oncoproteins interact with 14-3-3zeta in a PDZ binding motif-dependent manner. Journal of virology. 2013;87(3):1586–95. 10.1128/JVI.02074-12 23175360 PMC3554170

[50] Thomas M, Banks L. PDZRN3/LNX3 is a novel target of human papillomavirus type 16 (HPV-16) and HPV-18 E6. Journal of virology. 2015;89(2):1439–44. 10.1128/JVI.01743-14 25355882 PMC4300655

[51] Ajiro M, Zheng ZM. E6^E7, a novel splice isoform protein of human papillomavirus 16, stabilizes viral E6 and E7 oncoproteins via HSP90 and GRP78. MBio. 2015;6(1):e02068–14. 10.1128/mBio.02068-14 25691589 PMC4337564

[52] Kuang YQ, Charette N, Frazer J, Holland PJ, Attwood KM, Dellaire G, et al Dopamine receptor-interacting protein 78 acts as a molecular chaperone for CCR5 chemokine receptor signaling complex organization. PLoS One. 2012;7(7):e40522 10.1371/journal.pone.0040522 22815758 PMC3398031

[53] Boon SS, Tomaic V, Thomas M, Roberts S, Banks L. Cancer-causing human papillomavirus E6 proteins display major differences in the phospho-regulation of their PDZ interactions. Journal of virology. 2015;89(3):1579–86. 10.1128/JVI.01961-14 25410862 PMC4300763

[54] Liang YJ, Chang HS, Wang CY, Yu WC. DYRK1A stabilizes HPV16E7 oncoprotein through phosphorylation of the threonine 5 and threonine 7 residues. The international journal of biochemistry & cell biology. 2008;40(11):2431–41. 18468476 10.1016/j.biocel.2008.04.003

[55] Roman A, Munger K. The papillomavirus E7 proteins. Virology. 2013;445(1–2):138–68. 10.1016/j.virol.2013.04.013 23731972 PMC3783579

[56] Isaacson Wechsler E, Wang Q, Roberts I, Pagliarulo E, Jackson D, Untersperger C, et al Reconstruction of human papillomavirus type 16-mediated early-stage neoplasia implicates E6/E7 deregulation and the loss of contact inhibition in neoplastic progression. Journal of virology. 2012;86(11):6358–64. 10.1128/JVI.07069-11 22457518 PMC3372204

[57] Bellanger S, Demeret C, Goyat S, Thierry F. Stability of the human papillomavirus type 18 E2 protein is regulated by a proteasome degradation pathway through its amino-terminal transactivation domain. Journal of virology. 2001;75(16):7244–51. 10.1128/JVI.75.16.7244-7251.2001 11461997 PMC114960

[58] Buck CB, Thompson CD, Pang YY, Lowy DR, Schiller JT. Maturation of papillomavirus capsids. Journal of virology. 2005;79(5):2839–46. 10.1128/JVI.79.5.2839-2846.2005 15709003 PMC548454

[59] Wilson R, Ryan GB, Knight GL, Laimins LA, Roberts S. The full-length E1E4 protein of human papillomavirus type 18 modulates differentiation-dependent viral DNA amplification and late gene expression. Virology. 2007;362(2):453–60. 10.1016/j.virol.2007.01.005 17303206

[60] Davy C, McIntosh P, Jackson DJ, Sorathia R, Miell M, Wang Q, et al A novel interaction between the human papillomavirus type 16 E2 and E1—E4 proteins leads to stabilization of E2. Virology. 2009;394(2):266–75. 10.1016/j.virol.2009.08.035 19783272

[61] McKinney CC, Hussmann KL, McBride AA. The Role of the DNA Damage Response throughout the Papillomavirus Life Cycle. Viruses. 2015;7(5):2450–69. 10.3390/v7052450 26008695 PMC4452914

[62] Busillo JM, Benovic JL. Regulation of CXCR4 signaling. Biochim Biophys Acta. 2007;1768(4):952–63. 10.1016/j.bbamem.2006.11.002 17169327 PMC1952230

[63] McDermott DH, Liu Q, Velez D, Lopez L, Anaya-O'Brien S, Ulrick J, et al A phase 1 clinical trial of long-term, low-dose treatment of WHIM syndrome with the CXCR4 antagonist plerixafor. Blood. 2014;123(15):2308–16. 10.1182/blood-2013-09-527226 24523241 PMC3983611

[64] Meuris F, Gaudin F, Aknin ML, Hemon P, Berrebi D, Bachelerie F. Symptomatic Improvement in Human Papillomavirus-Induced Epithelial Neoplasia by Specific Targeting of the CXCR4 Chemokine Receptor. The Journal of investigative dermatology. 2016;136(2):473–80. 10.1016/j.jid.2015.11.004 26967480

[65] Bachelerie F, Ben-Baruch A, Burkhardt AM, Combadiere C, Farber JM, Graham GJ, et al International Union of Pharmacology. LXXXIX. Update on the extended family of chemokine receptors and introducing a new nomenclature for atypical chemokine receptors. Pharmacol Rev. 2014;66(1):1–79. 10.1124/pr.113.007724 24218476 PMC3880466

[66] Freitas C, Desnoyer A, Meuris F, Bachelerie F, Balabanian K, Machelon V. The relevance of the chemokine receptor ACKR3/CXCR7 on CXCL12-mediated effects in cancers with a focus on virus-related cancers. Cytokine & growth factor reviews. 2014;25(3):307–16. 24853339 10.1016/j.cytogfr.2014.04.006

[67] Teicher BA, Fricker SP. CXCL12 (SDF-1)/CXCR4 pathway in cancer. Clinical cancer research: an official journal of the American Association for Cancer Research. 2010;16(11):2927–31. 20484021 10.1158/1078-0432.CCR-09-2329

[68] Kryczek I, Wei S, Keller E, Liu R, Zou W. Stroma-derived factor (SDF-1/CXCL12) and human tumor pathogenesis. Am J Physiol Cell Physiol. 2007;292(3):C987–95. 10.1152/ajpcell.00406.2006 16943240

[69] Burger JA, Kipps TJ. CXCR4: a key receptor in the crosstalk between tumor cells and their microenvironment. Blood. 2006;107(5):1761–7. 10.1182/blood-2005-08-3182 16269611

[70] Treon SP, Xu L, Yang G, Zhou Y, Liu X, Cao Y, et al MYD88 L265P somatic mutation in Waldenstrom's macroglobulinemia. N Engl J Med. 2012;367(9):826–33. 10.1056/NEJMoa1200710 22931316

[71] Balabanian K, Levoye A, Klemm L, Lagane B, Hermine O, Harriague J, et al Leukocyte analysis from WHIM syndrome patients reveals a pivotal role for GRK3 in CXCR4 signaling. The Journal of clinical investigation. 2008;118(3):1074–84. 10.1172/JCI33187 18274673 PMC2242619

[72] Pasquet M, Bellanne-Chantelot C, Tavitian S, Prade N, Beaupain B, Larochelle O, et al High frequency of GATA2 mutations in patients with mild chronic neutropenia evolving to MonoMac syndrome, myelodysplasia, and acute myeloid leukemia. Blood. 2013;121(5):822–9. 10.1182/blood-2012-08-447367 23223431 PMC3714670

[73] Maciejewski-Duval A, Meuris F, Bignon A, Aknin ML, Balabanian K, Faivre L, et al Altered chemotactic response to CXCL12 in patients carrying GATA2 mutations. J Leukoc Biol. 2015 26710799 10.1189/jlb.5MA0815-388R

