# Supplementary figures and images for: The CXCL12/CXCR4 Signaling Pathway: A New Susceptibility Factor in Human Papillomavirus Pathogenesis
Source: PLoS Pathog. 2016 Dec 5;12(12):e1006039. doi: 10.1371/journal.ppat.1006039 (PMC5138052; doi:10.1371/journal.ppat.1006039)

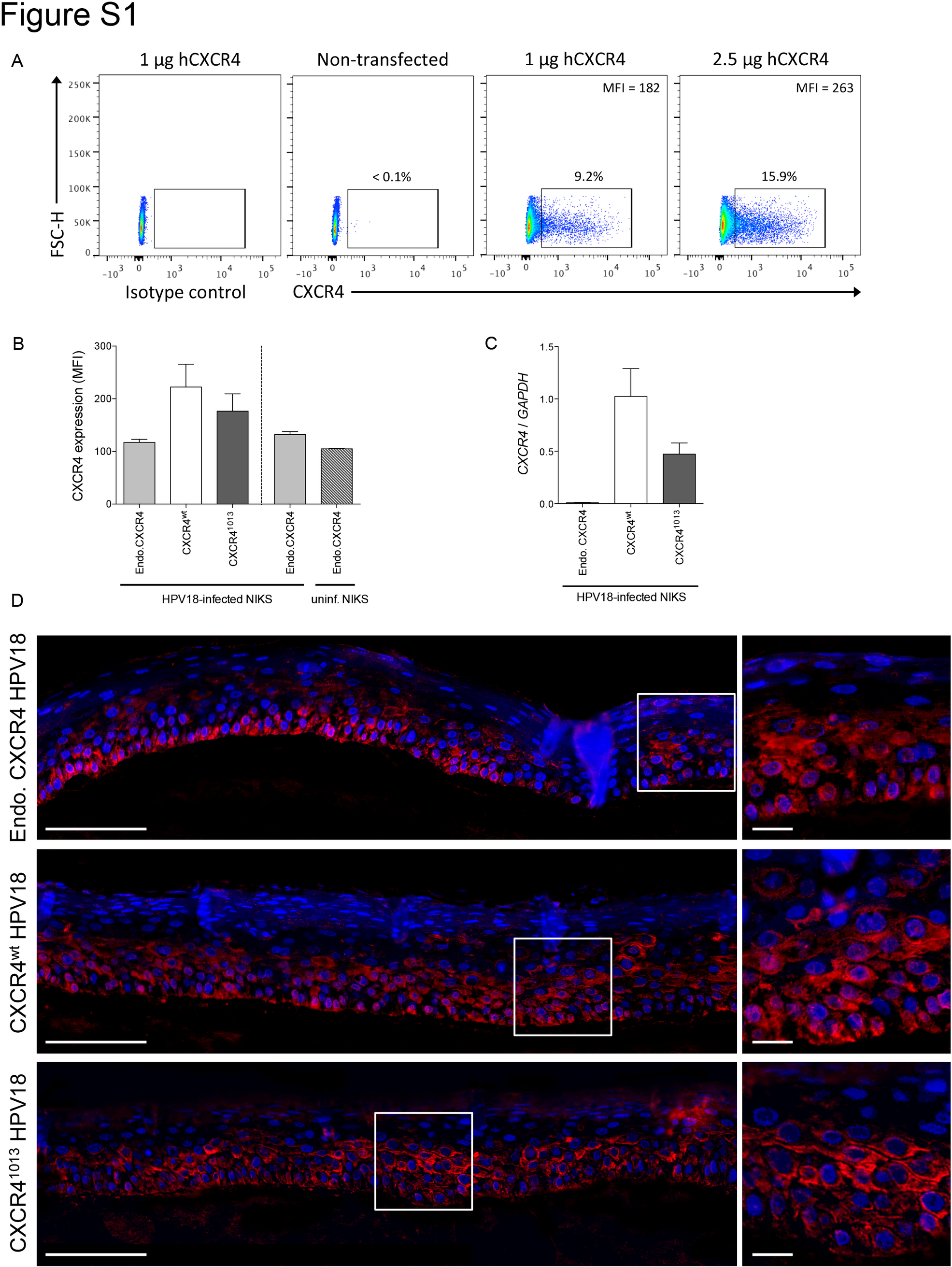

Supplement: S1 Fig — (A) Cell surface expression of CXCR4 in CHO cells transfected with 1 and 2.5 μg of a vector encoding for the human CXCR4 receptor was investigated by flow cytometry using the 12G5 antibody, which specifically recognizes the human form of CXCR4 and not the endogenous Chinese hamster CXCR4 receptor. (B) Cell surface expression of CXCR4 in HPV18-positive NIKS cells, non-transduced (i.e. endogenous (Endo.) CXCR4) as compared to cells transduced with lentiviral vectors expressing CXCR4wt or CXCR41013 (left graph) or to uninfected NIKS cells (right graph). Cell surface expression of CXCR4, investigated by flow cytometry using the 12G5 antibody, is represented as mean fluorescence intensity (MFI) ± SEM (n = 3). (C) HPV18-positive NIKS cells non-transduced (i.e. endogenous CXCR4) or transduced with lentiviral vectors expressing CXCR4wt or CXCR41013 were investigated for CXCR4 transcripts levels. Transcripts were expressed as relative levels normalized to GAPDH transcripts levels (mean ± SEM, n = 3). (D) Detection of CXCR4 expression by immunofluorescence in HPV18-positive raft cultures sections (i.e. endogenous CXCR4) and in HPV18-positive CXCR4wt and CXCR41013 raft culture sections. Images are representative of three independent experiments. Scale bars = 100 μm, inset scale bars = 20 μm. (TIF) [file ppat.1006039.s001.tif]

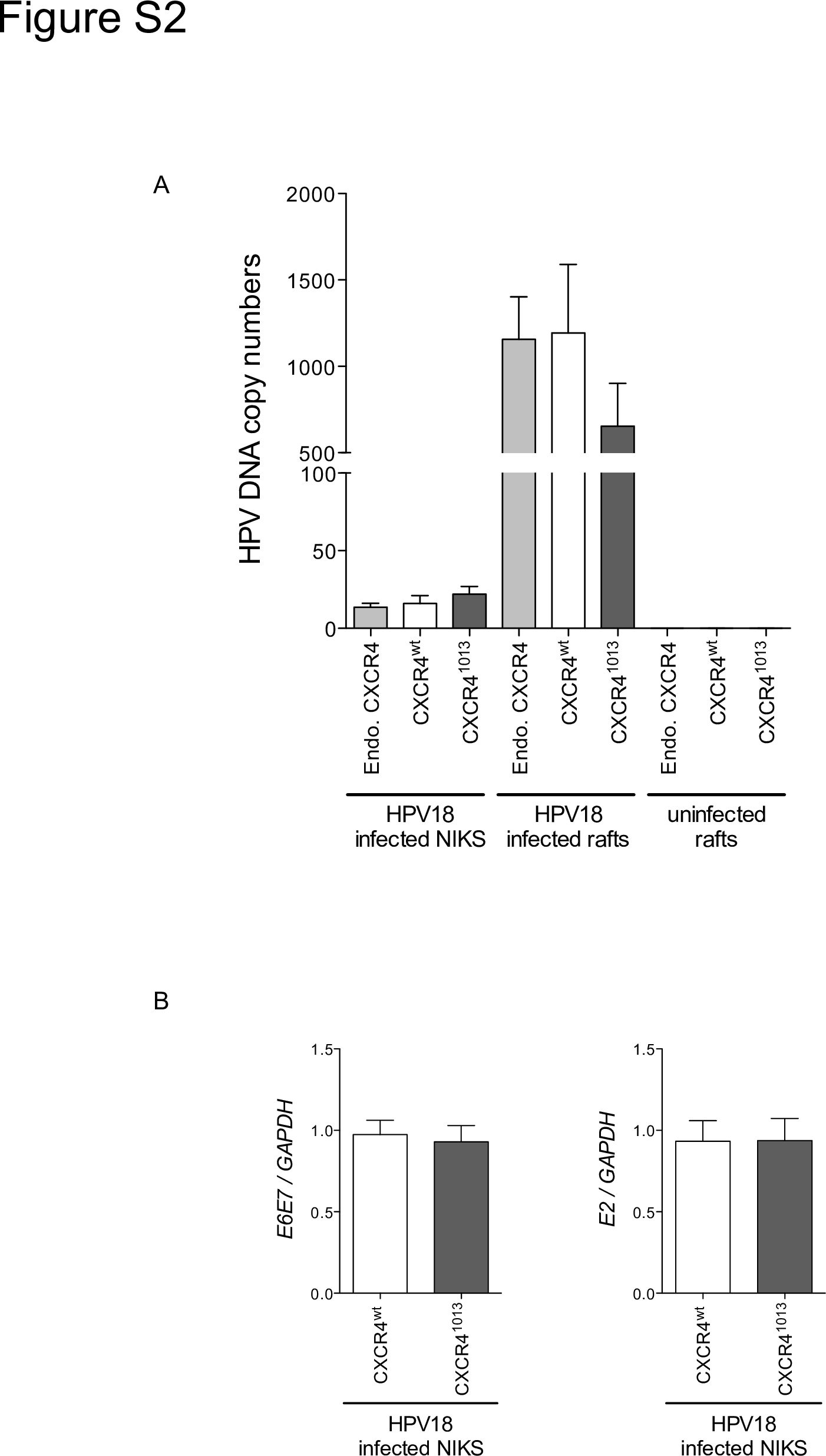

Supplement: S2 Fig — (A) HPV18-positive NIKS cells non-transduced (i.e. endogenous CXCR4) or transduced with lentiviral vectors expressing CXCR4wt or CXCR41013 were investigated for HPV18 DNA copy numbers before (i.e. HPV18-infected NIKS) and after being differentiated into 3D cultures (i.e. HPV18-infected rafts). Uninfected rafts were also integrated as negative control. HPV18 DNA copy numbers are expressed as the ratio to GAPDH gene copy numbers (mean ± SEM, n = 3). (B) HPV18-E6/E7 and HPV18-E2 transcripts levels in HPV18-positive CXCR4wt and CXCR41013 NIKS cells cultured in monolayers before being differentiated into raft cultures (see S6 Fig). Transcripts were expressed as relative levels normalized to GAPDH transcripts levels (mean ± SEM, n = 3). (TIF) [file ppat.1006039.s002.tif]

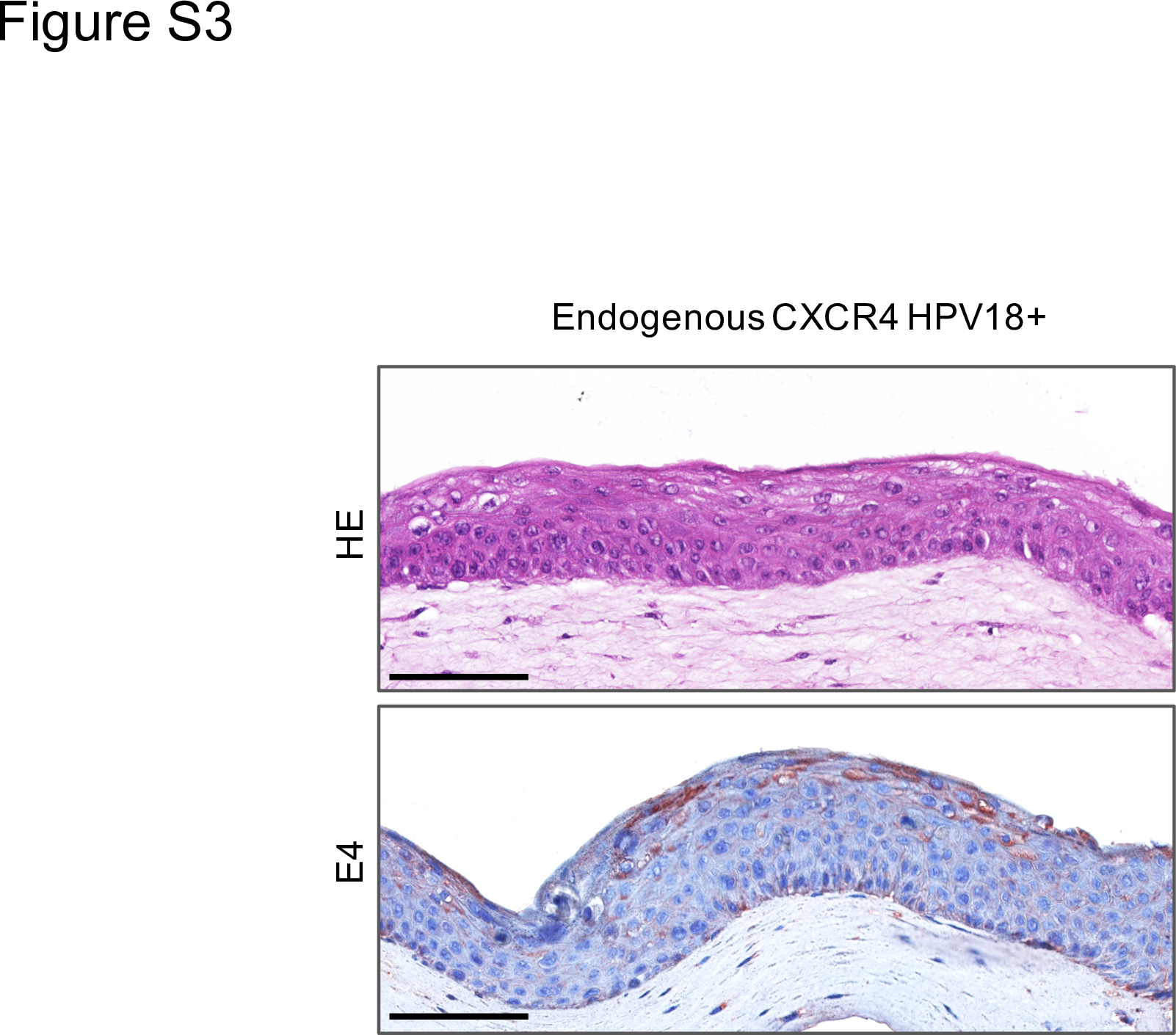

Supplement: S3 Fig — Representative section of HPV18-positive raft cultures stained with hematoxylin and eosin (HE; upper panel) and for HPV18-E4 protein (lower panel). Images are representative of three independent experiments. Scale bars = 100 μm. (TIF) [file ppat.1006039.s003.tif]

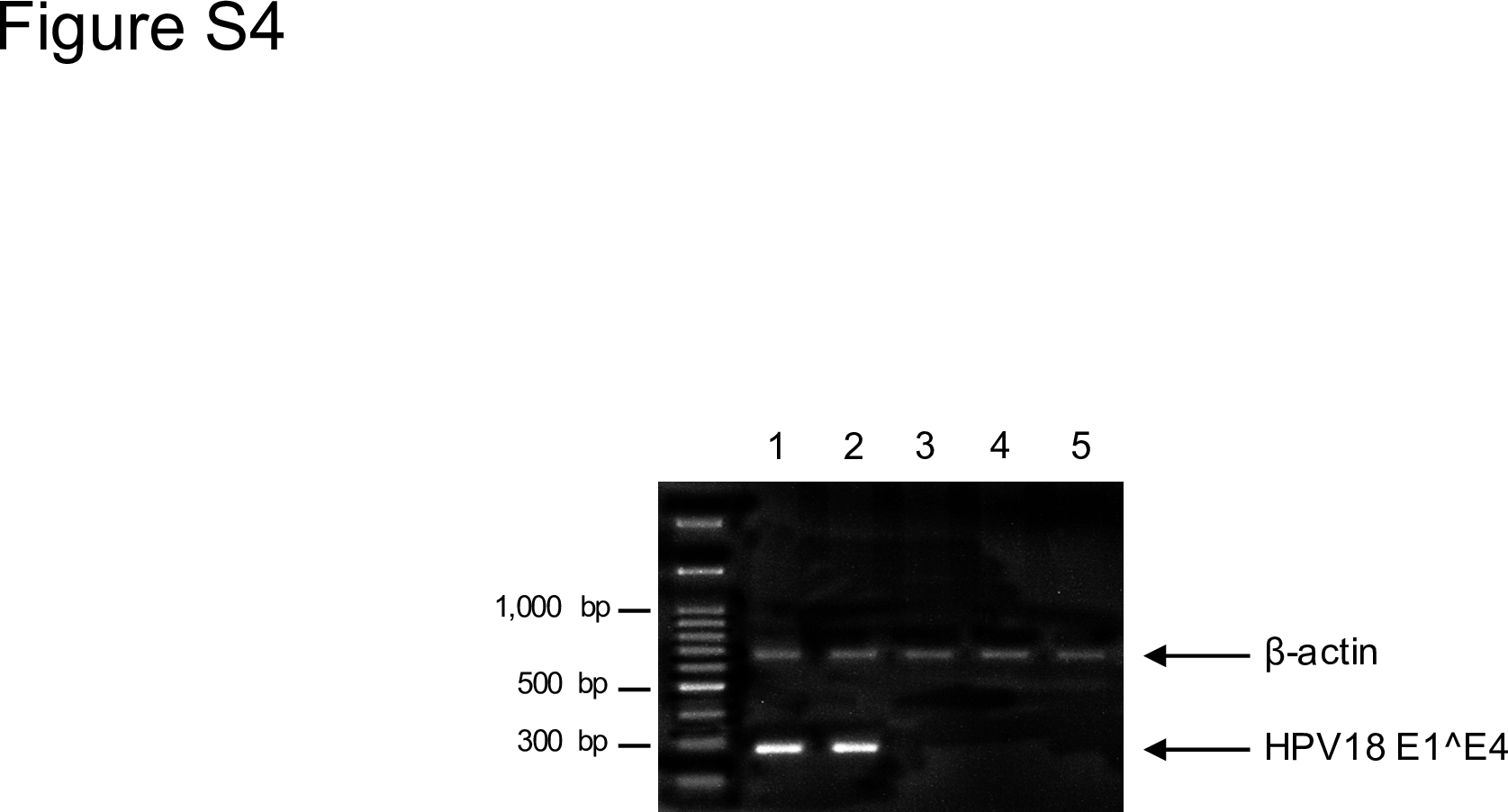

Supplement: S4 Fig — HaCat cells were infected with a 1:20 or 1:100 dilution of viral stocks harvested from either HPV18-positive CXCR4wt or CXCR41013 raft cultures. Shown is a 2% agarose gel of nested RT-PCR-amplified β-actin and HPV18 E1^E4. Lane 1, CXCR4wt HPV18 at 1:20. Lane 2, CXCR4wt HPV18 at 1:100. Lane 3, CXCR41013 HPV18 at 1:20. Lane 4, CXCR41013 HPV18 at 1:100. Lane 5, negative control (no virus). β-actin and HPV18 E1^E4 sequences were confirmed by sequencing and positions are indicated in the right and molecular size markers are indicated in the left. (TIF) [file ppat.1006039.s004.tif]

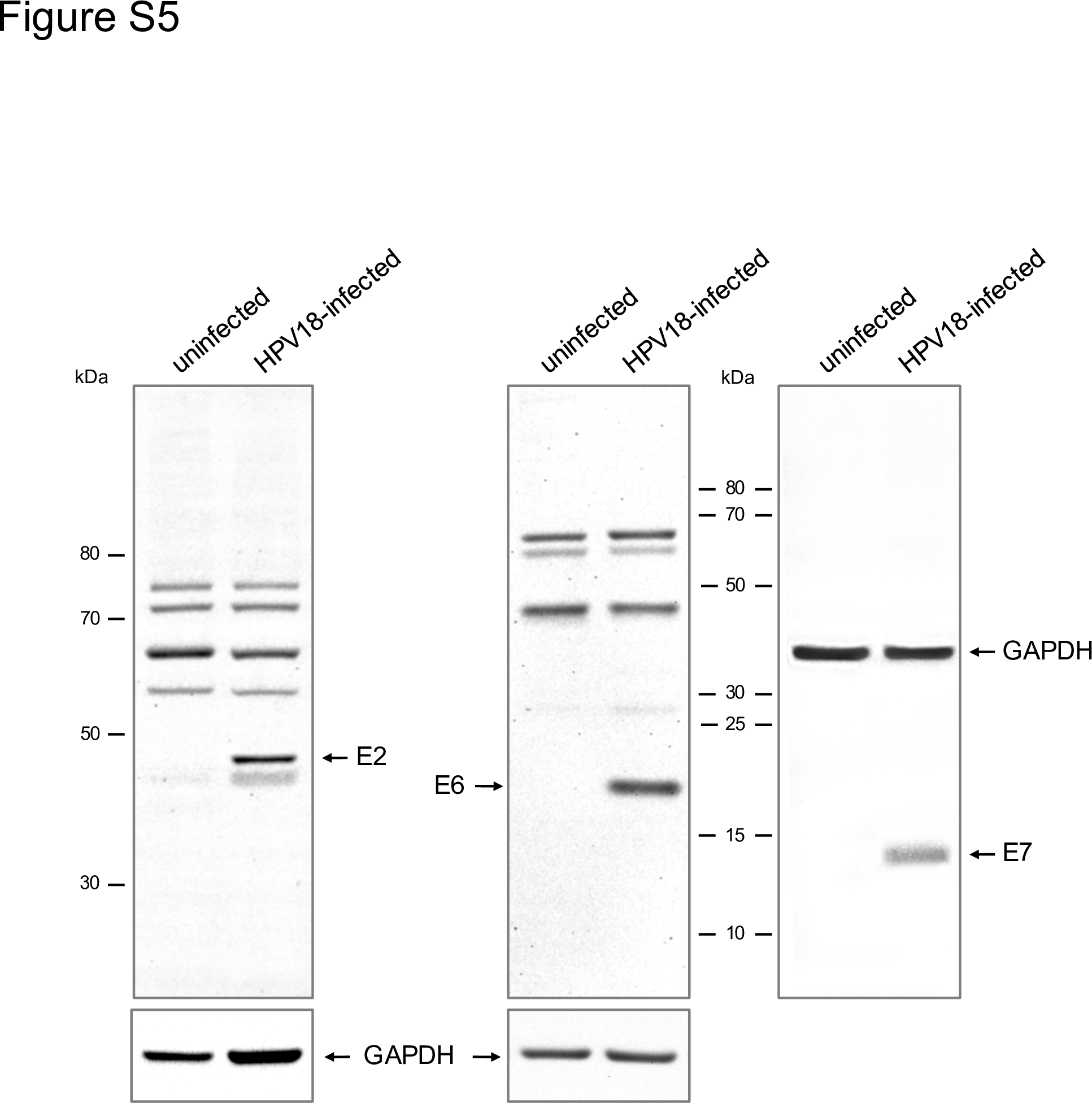

Supplement: S5 Fig — Western blots showing detection of HPV18-E2, HPV18-E6 and HPV18-E7 proteins in uninfected (negative control for the detection of HPV18 proteins) versus HPV18-infected conditions (rafts or NIKS cells). Proteins were extracted from raft cultures (left panel) or NIKS cells (central and right panels). GAPDH detection and size markers are also shown. (TIF) [file ppat.1006039.s005.tif]

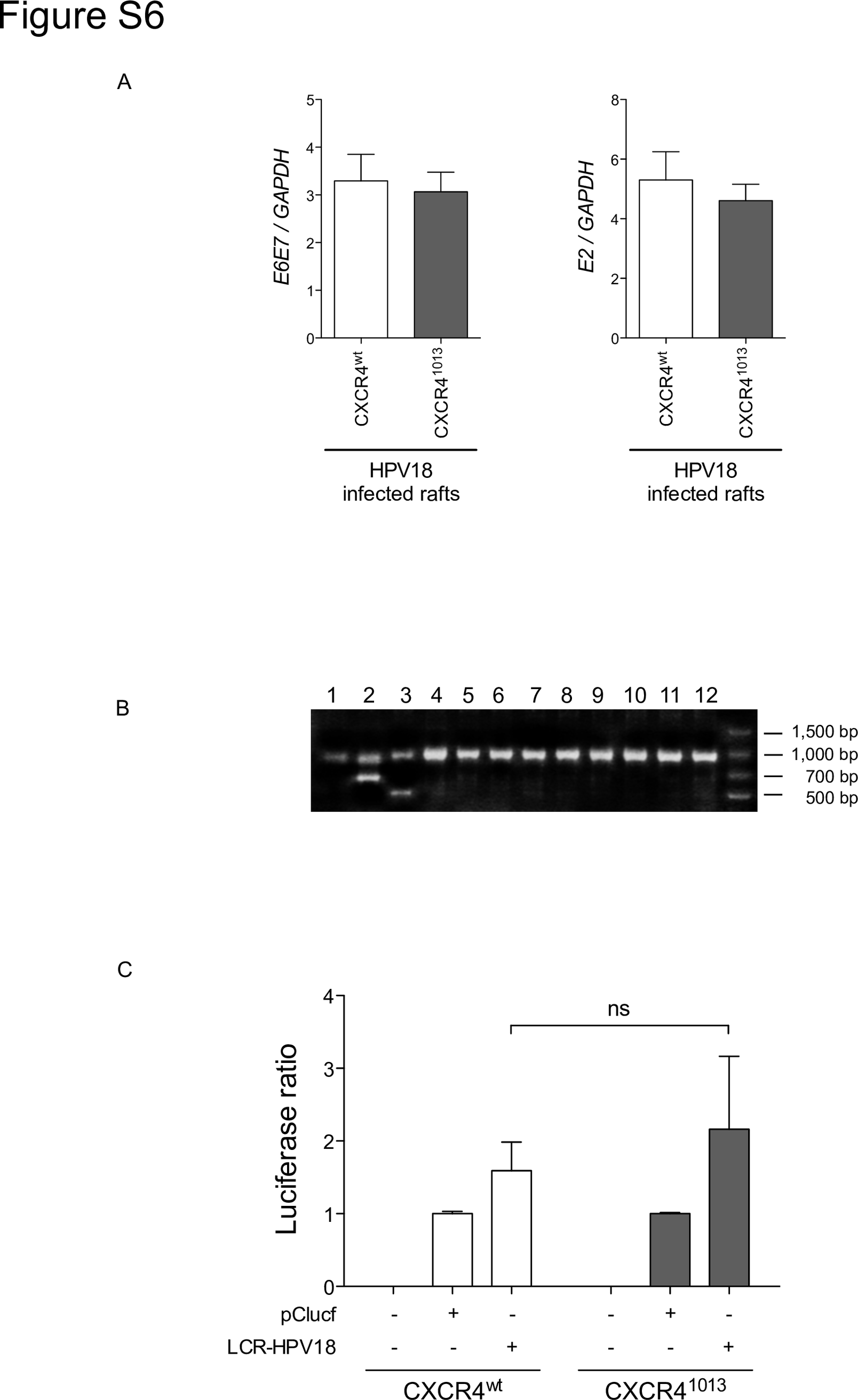

Supplement: S6 Fig — HPV18-positive CXCR4wt and CXCR41013 raft cultures were investigated (A) for HPV18-E6/E7 and HPV18-E2 transcripts levels (transcripts were expressed as relative levels normalized to GAPDH transcripts levels (mean ± SEM, n = 3)), and (B) for HPV integration using the APOT assay. Shown is a 1.2% agarose gel of nested RT-PCR-amplified HPV E7. Lane 1, negative control (HaCat cells); lanes 2 and 3, positive controls (Human keratinocytes and HeLa cells, respectively, containing integrated HPV18 genome); lanes 4 to 6, HPV18-infected NIKS, CXCR4wt NIKS and CXCR41013 NIKS, respectively; lanes 7 and 10, HPV18 infected NIKS-derived rafts; lanes 8 and 11, CXCR4wt-rafts; Lanes 9 and 12, CXCR41013-rafts. Molecular size markers are indicated in the right and positive controls in lanes 2 and 3 were confirmed by sequencing. (C) Luciferase reporter assays was used to investigate the intrinsic promoter activity of the HPV18 LCR in NIKS cells transduced for expression of CXCR4wt or CXCR41013, and transiently transfected with the LCR-HPV18-luciferase vector. Luciferase ratio represents the fold increase of luciferase signal over the luciferase activity in cells transfected with the control pClucF plasmid (mean ± SEM, n = 3). (TIF) [file ppat.1006039.s006.tif]

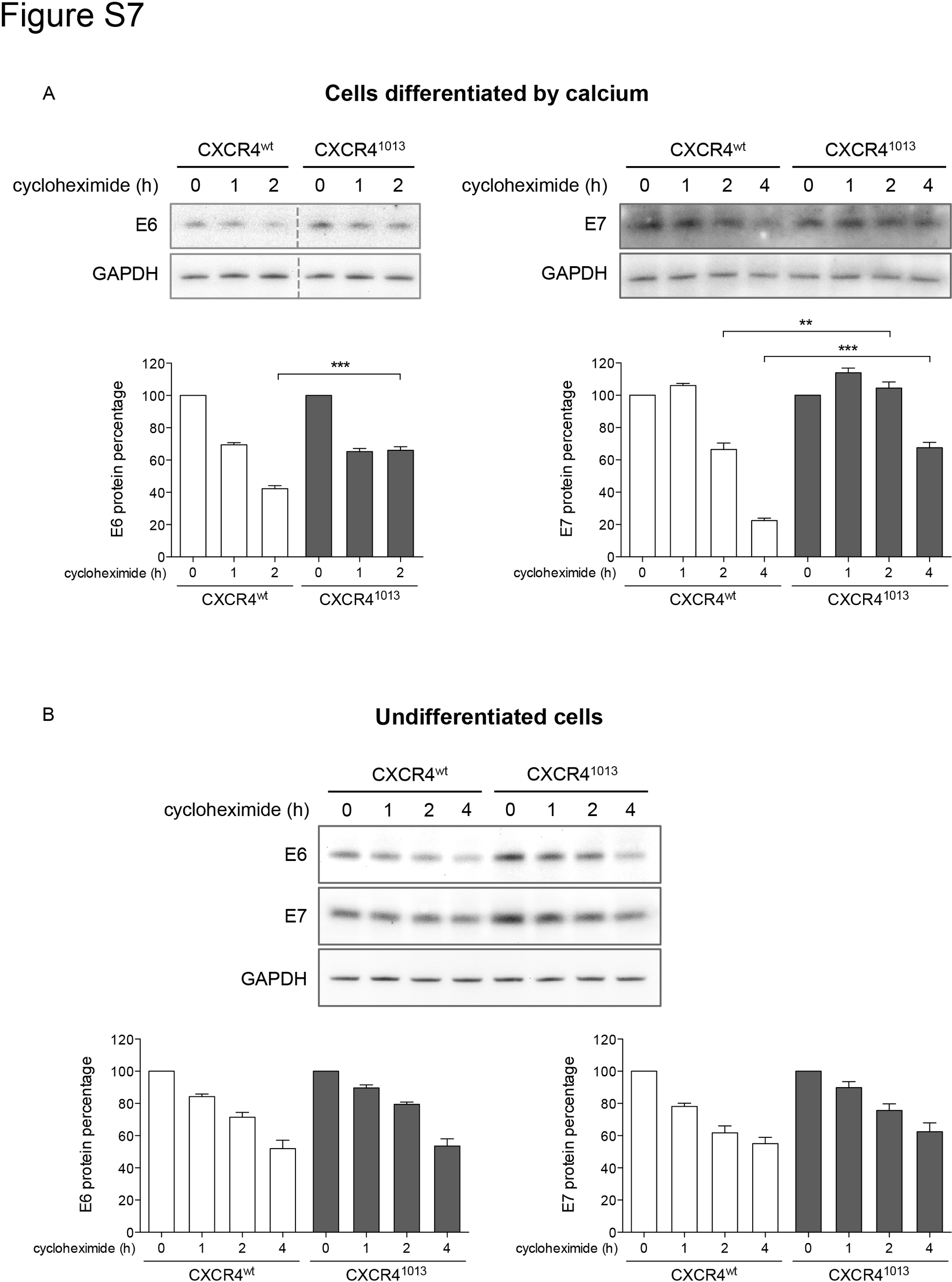

Supplement: S7 Fig — Western blots (upper panels) and densitometric analyses (lower panels) showing relative levels of HPV18-E6 and HPV18-E7 in HPV18-positive CXCR4wt and CXCR41013 NIKS cells differentiated in high-calcium media for 96 h (A) or undifferentiated (B). NIKS cells were treated with cycloheximide (50 μg/mL) for the indicated times. Densitometric analyses represent E6 and E7 protein levels normalized to GAPDH protein levels and expressed as percent of the initial levels at time 0 set at 100%. (A) E6 protein levels were 0.19 +/- 0.01; 0.14 +/- 0.01; 0.086 +/- 0.008 and 0.25 +/- 0.01; 0.167 +/- 0.01; 0.168 +/- 0.01 (at 0, 1 and 2 h post cycloheximide for CXCR4wt and CXCR41013, respectively). E7 protein levels were 2.0 +/- 0.12; 2.13 +/- 0.10; 1.34 +/- 0.16; 0.44 +/- 0.01 and 1.18 +/- 0.029; 1.34 +/- 0.019; 1.23 +/- 0.023; 0.79 +/- 0.03 (0, 1, 2 and 4 h post cycloheximide for CXCR4wt and CXCR41013, respectively). (B) E6 protein levels were 0.56 +/- 0.10; 0.48 +/- 0.10; 0.41 +/- 0.10; 0.30 +/- 0.09 and 0.76 +/- 0.09; 0.71 +/- 0.11; 0.64 +/- 0.10; 0.43 +/- 0.11 (0, 1, 2 and 4 h post cycloheximide for CXCR4wt and CXCR41013, respectively). E7 protein levels were 0.3 +/- 0.08; 0.24 +/- 0.07; 0.19 +/- 0.07; 0.17 +/- 0.06 and 0.42 +/- 0.09; 0.38 +/- 0.10; 0.32 +/- 0.09; 0.27 +/- 0.09 (0, 1, 2 and 4 h post cycloheximide for CXCR4wt and CXCR41013, respectively). Values are the mean ± SEM. **p < 0.01 and ***p < 0.001. (TIF) [file ppat.1006039.s007.tif]

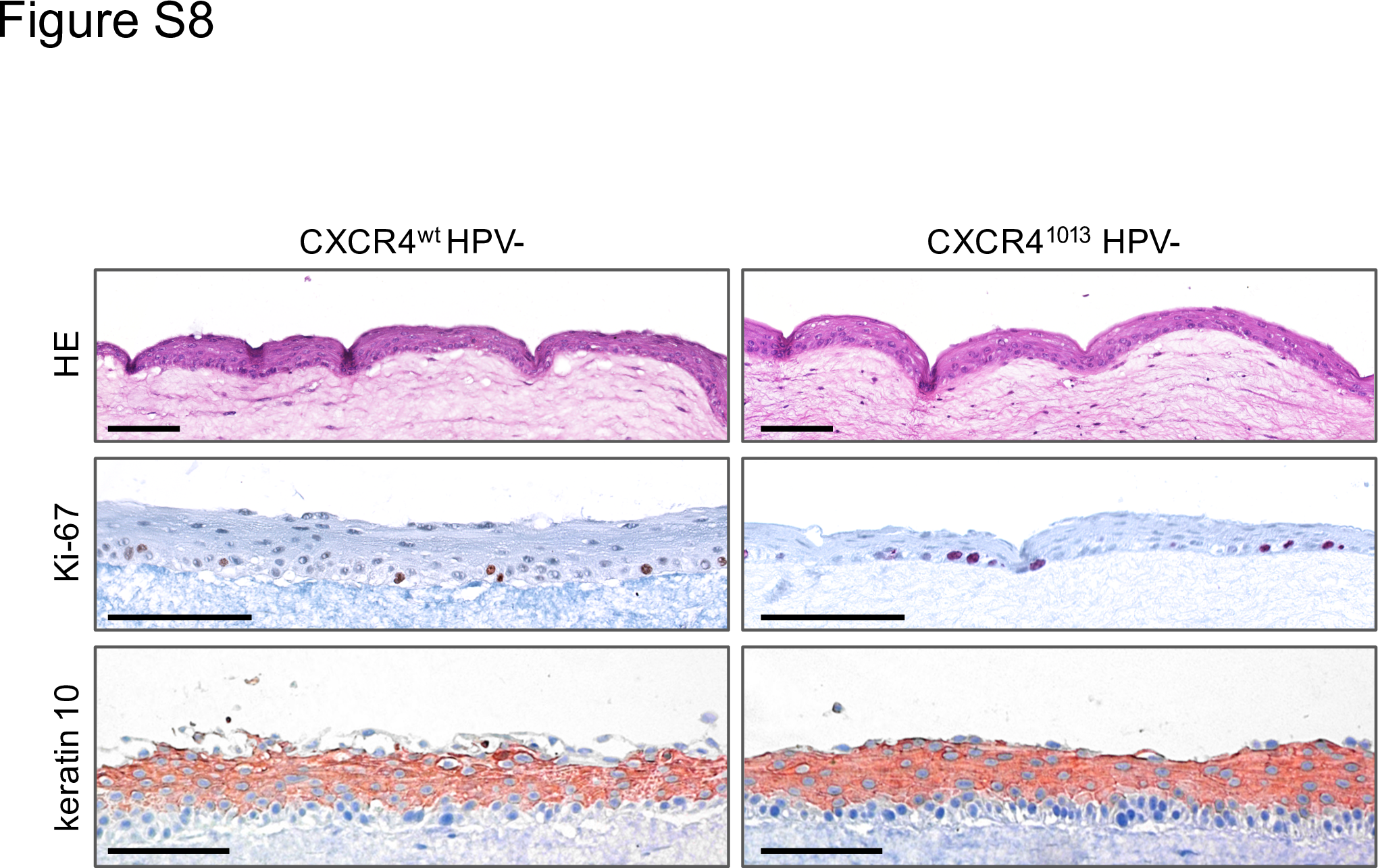

Supplement: S8 Fig — Representative sections of HPV-negative (HPV-) CXCR4wt and CXCR41013 raft cultures stained with hematoxylin and eosin (HE; upper panel), or for Ki-67 or keratin 10 expression (middle and lower panel, respectively). Images are representative of three independent experiments. Scale bars = 100 μm. (TIF) [file ppat.1006039.s008.tif]

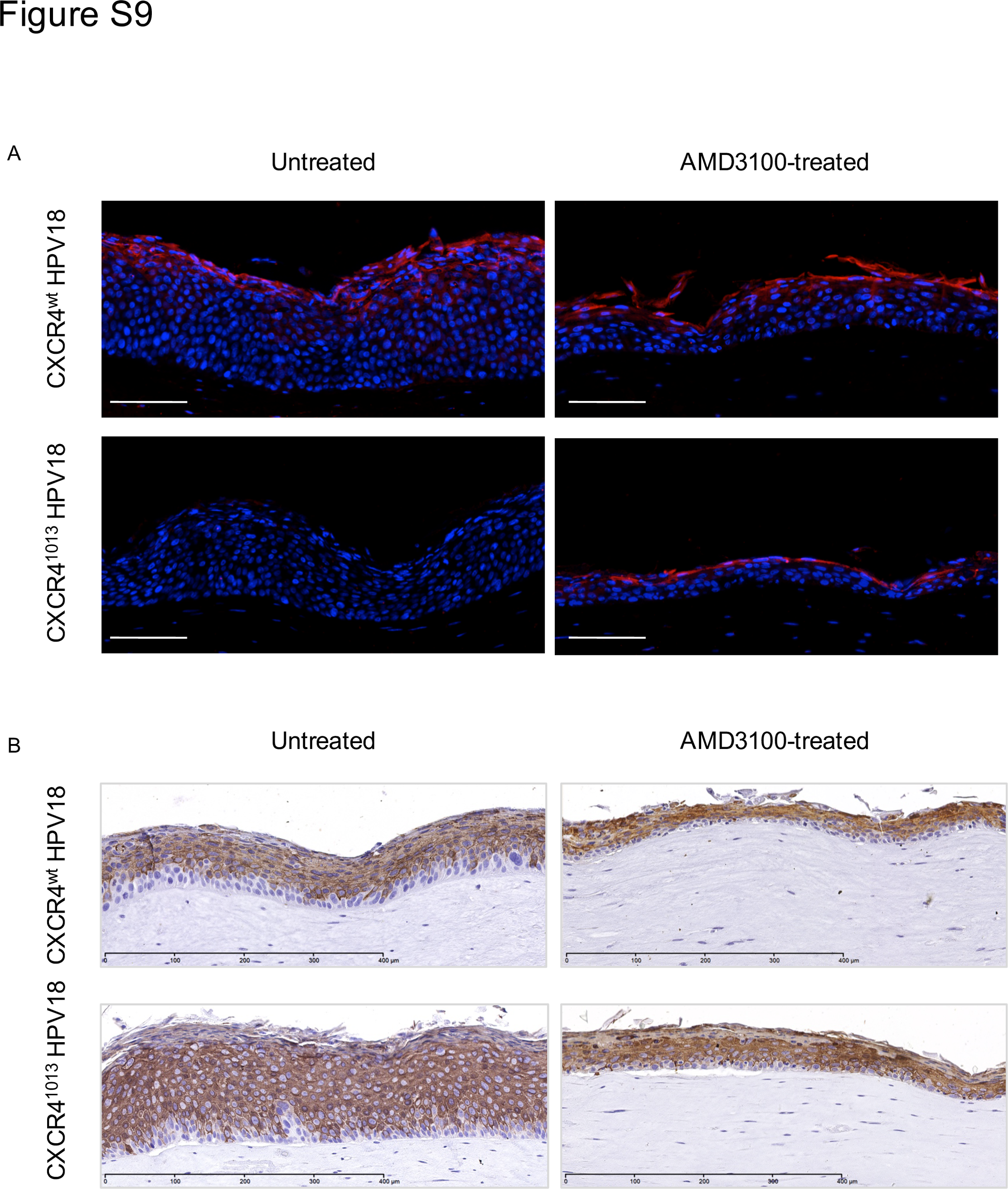

Supplement: S9 Fig — Representative sections of HPV18-positive CXCR4wt and CXCR41013 raft cultures treated (AMD3100-treated) or not (untreated) with AMD3100 were investigated (A) for keratin 10, and (B) for HPV18-E4 expression. Images are representative of three independent experiments. Scale bars = 100 μm (A) and as shown (B). (TIF) [file ppat.1006039.s009.tif]

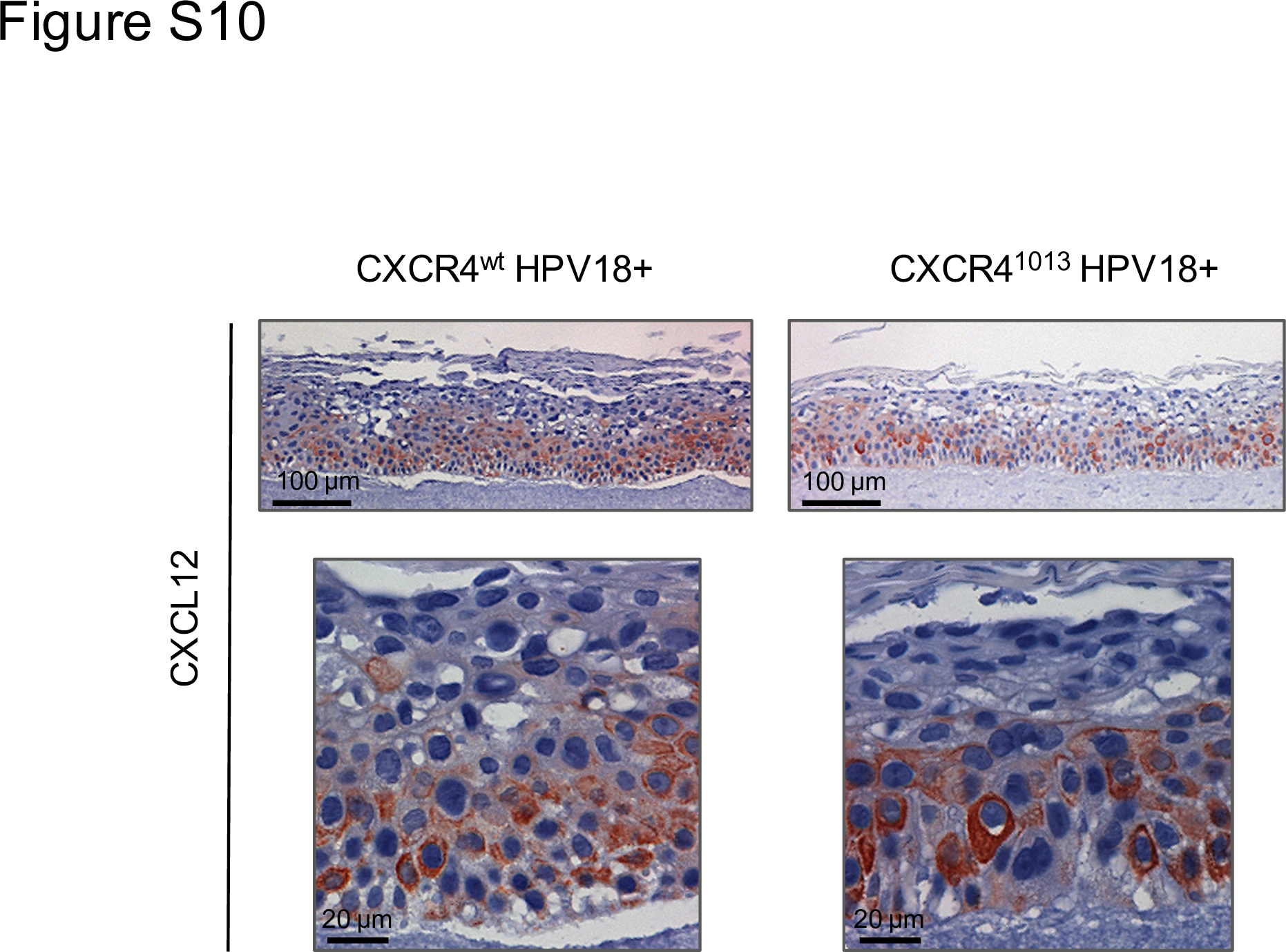

Supplement: S10 Fig — Representative sections of HPV18-positive CXCR4wt and CXCR41013 raft cultures stained for CXCL12 protein. Immunohistochemistry was performed using primary antibody for CXCL12. (TIF) [file ppat.1006039.s010.tif]
