# Supplementary material for: The CXCL12/CXCR4 Signaling Pathway: A New Susceptibility Factor in Human Papillomavirus Pathogenesis
Source: PLoS Pathog. 2016 Dec 5;12(12):e1006039. doi: 10.1371/journal.ppat.1006039 (PMC5138052; doi:10.1371/journal.ppat.1006039)
Supplement: S1 Protocol — HPV detection by quantitative real-time PCR analysisLuciferase assayAPOT assayVirus isolation and in vitro infectivity essayExpression of CXCR4 by flow cytometry, quantitative real-time PCR and immunofluorescence (DOCX) [file ppat.1006039.s011.docx]

**PROTOCOL S1**

**A. HPV detection by quantitative real-time PCR analysis**

Total RNA from HPV18-infected NIKS cells was extracted with the RNeasy Mini Kit (QIAGEN), while total RNA from raft samples was extracted with the RNeasy Fibrous Tissue Mini kit (QIAGEN). Total RNA was reverse transcribed with the SuperScript III Reverse Transcriptase (Invitrogen). Real-time PCR was performed on a LightCycler® 480 System, using the LightCycler® 480 Probes Master (Roche Life Science). HPV18-E6/E7: 5'-CACAATGTTGTGTATGTGTTGTAAGTG-3' (forward), 5'-GGTCGTCTGCTGAGCTTTCTA-3' (reverse), Universal ProbeLibrary probe #129. HPV18-E2: 5'-GGGAACATGGCATACAGACA-3' (forward), 5'-GCCATTTGCAGTTCAATAGCTT-3' (reverse), Universal ProbeLibrary probe #131. GAPDH: Universal ProbeLibrary Human GAPD Gene Assay (Roche Life Science). Total DNA was extracted with the DNeasy Blood & Tissue kit (QIAGEN). HPV18 DNA copies were detected using the TaqMan Universal PCR Master Mix (Applied Biosystems). HPV18: 5’-CCAGACGTCGGCTGCTACA-3’ (forward), 5’-GACAGGTCCACAATGCTGCTT-3’ (reverse), 5’-CCTGGACACTGTGGACTCGCGGA-3’ (probe). GAPDH: 5’-CCTCCCGCTTCGCTCTCT-3’ (forward), 5’-CTGGCGACGCAAAAGAAGA-3’ (reverse), 5’-TCCTCCTGTTCGACAGTCAGCCGC-3’ (probe).

**B. Luciferase assay**

NIKS cells expressing either CXCR4^wt^ or CXCR4^1013^ were transiently transfected with 2 μg of LCR-HPV18 (encompassing the complete HPV18’ LCR region upstream the luciferase gene) or pClucf DNA (encompassing the luciferase gene downstream the CMV promoter) (mixed with 2,5 M CaCl_2_ solution, diluted in HEPES buffered saline) in F medium supplemented with 10% HEPES during 7 h. Cells were plated in a 6 wells plate (1.10^5^ 3T3 cells and 6.10^5^ NIKS) and the luciferase assay (Promega kit) was done 48 h after transfection using a Mithras LB 940 luminometer (Berthold Technologies). The BCA Protein Assay System kit (Thermo Scientific) was used for the quantification of total protein. Relative luciferase activity measurements were normalized to total protein concentration. Averages were based on the mean of duplicates of three independent experiments.

**C. APOT assay**

Integration of the HPV18 genome was investigated by the APOT assay performed as previously described (Klaes R. et al., Cancer Res. 1999, PMID: 10626803). Briefly, total RNA was extracted from raft samples with the RNeasy Fibrous Tissue Mini kit (QIAGEN). Reverse transcription was performed using an adaptor linked oligo(dT)-primer (dT)17-p3, followed by a semi-nested PCR using HPV18 E7 specific primers P1-HPV18 and P3 for the first PCR, and P2-HPV18 and (dT)17-p3 for the nested PCR. The final PCR products were electrophoresed in 1.2% agarose gels. All fusion transcripts were confirmed by direct sequencing of amplification products. (dT)17-p3: 5’-GACTCGAGTCGACATCGATTTTTTTTTTTTTTTTT-3’, P1-HPV18: 5’-TAGAAAGCTCAGCAGACGACC-3’, P3: 5’-GACTCGAGTCGACATCG-3’, P2-HPV18: 5’-ACGACCTTCGAGCATTCCAGCAG-3’.

**D. Virus isolation and in vitro infectivity assay**

HPV18 virions were isolated from the CXCR4^wt^- or CXCR4^1013^-derived raft cultures as described (McLaughlin-Drubin ME. et al., Methods Mol Med. 2005, PMID:16350404), resuspended in 0.5 mL of phosphate-buffered saline and stored at -20°C. The HPV18 infectivity studies were done in the HaCat cell line based on the in vitro system previously described (Smith LH. et al., J Invest Dermatol. 1995, PMID:7665926). HaCat were cultured in Dulbecco's Modified Eagle Medium GlutaMAX^TM^ (61965-026, Gibco) containing 10% fetal bovine serum and 1% penicillin-streptomycin and plated in a 24-well plate one day prior to infection. The HPV18 virus stocks were passed six times through a 21 gauge needle, diluted to 1:20 and 1:100 with cell culture medium and added to the cells for 48 h at 37°C. Wells receiving 0.5 mL of medium without virus were used as control. Efficiency of infection was measured via the detection of spliced HPV18 E1^E4 mRNA species as previously described. Briefly, mRNA was extracted from HaCat cells by using the mRNA Capture Kit (11787896001, Roche Life Science) as described by the manufacturer. The mRNA captured in the streptavidin-coated PCR tubes was used in a RT reaction utilizing reagents from the SuperScript® III First-Strand Synthesis Supermix kit (18080-400, Life Technologies). The resulting cDNA was used for nested PCR to detect the HPV18 E1^E4 cDNA. A set of primers specific for β-actin was also included in the PCR mixture as a control for the quantification of cellular mRNA. The first PCR reaction was performed on the cDNA using a first set of primers containing HPV18-F1, HPV18-R1, βactin-F1, and βactin-R1, with the following temperature profile: 95°C for 5 min, followed by 40 cycles of 95°C for 30 s, 60°C for 30 s, 72°C for 1 min with a final 10-min extension at 72°C. 10% of the first PCR mixture was used as template for the nested reaction. This second PCR reaction was performed using a second set of primers containing HPV18-F2, HPV18-R2, βactin-F2, and βactin-R2, with the following temperature profile: 95°C for 5 min, followed by 40 cycles of 95°C for 30 s, 60°C for 30 s, 72°C for 30 sec with a final 10-min extension at 72°C. All PCR reactions were performed using the *Taq* DNA Polymerase recombinant kit (10342-020, Life Technologies) and contained 1X PCR Buffer -Mg, 2.5 mM MgCl_2_, 200 µM dNTPs, 125 ng of each forward and reverse primers, and 2.5 units of Taq polymerase. All PCR products were visualized by electrophoresis in a 2% agarose-ethidium bromide gel. HPV18-F1: 5’-GTTGTGTATGTGTTGTAAGTGTGA-3’, HPV18-R1: 5’-GTCCACAATGCTGCTTCTCCG-3’, HPV18-F2: 5’-GAATTGGCTAGTAGTAGAAAGCT-3’, HPV18-R2: 5’-TCCCACGTGTCCAGGTCGTGT-3’, βactin-F1: 5’-GAACCCCAAGGCCAACCGCGA-3’, βactin-R1: 5’-CCACACAGAGTACTTGCGCTCAGG-3’, βactin-F2: 5’-GATGACCCAGATCATGTTTG-3’, βactin-R2: 5’-GGAGCATGATCTTGATCTTC-3’.

**E. Expression of CXCR4 by flow cytometry, quantitative real-time PCR and immunofluorescence**

CXCR4 expression was investigated by flow cytometry using the 12G5 mAb (PE anti-CXCR4 12G5, BD Bioscience), a conformational antibody, which is specific for the human CXCR4 receptor. As control for antibody specificity, we used CHO (Chinese hamster ovary) cells that were transfected by a vector encoding for the human CXCR4 using the lipofectamine reagent according to the manufacturer recommendation and analyzed 48h later for CXCR4 expression. Analyses of CXCR4 transcripts in the indicated cell populations were performed by quantitative real-time PCR on a LightCycler® 480 System, using the LightCycler® 480 SYBR Green I Master (Roche Life Science). CXCR4: 5'-GACCGCTACCTGGCCATC-3' (forward), 5'-GGCAGCCAACAGGCGAAGA-3' (reverse). GAPDH: 5’-AATCCCATCACCATCTTCCA-3’ (forward), 5’-TGGACTCCACGACGTACTCA-3’ (reverse). The detection of CXCR4 expression at the protein level in raft cultures was investigated by immunofluorescence on paraffin sections. Staining with the primary antibody 12G5 (5 μg/mL, eBioscience) was followed by staining with a goat anti-mouse Alexa Fluor 594 (Invitrogen). Tissues were counterstained with DAPI.
